# Vascular dysfunction occurs prior to the onset of amyloid pathology and Aβ plaque deposits colocalize with endothelial cells in the hippocampus of female APPswe/PSEN1dE9 mice

**DOI:** 10.1007/s11357-024-01213-0

**Published:** 2024-06-11

**Authors:** Emily W. Waigi, Laena Pernomian, Alexia M. Crockett, Tiago J. Costa, Paul Townsend, R. Clinton Webb, Joseph A. McQuail, Cameron G. McCarthy, Fiona Hollis, Camilla F. Wenceslau

**Affiliations:** 1 Cardiovascular Translational Research Center, Department of Cell Biology and Anatomy, University of South Carolina School of Medicine, Columbia, SC USA; 2 Department of Pharmacology, Physiology and Neuroscience, University of South Carolina School of Medicine, Columbia, SC USA; 3 Department of Biomedical Engineering, College of Engineering and Computing, University of South Carolina, Columbia, SC USA

**Keywords:** Amyloid β_1-42_, Alzheimer’s disease, Vascular dysfunction, APPswe/PSEN1dE9 mice, Vascular stiffness

## Abstract

Increasing evidence shows that cardiovascular diseases (CVDs) are associated with an increased risk of cognitive impairment and Alzheimer’s diseases (AD). It is unknown whether systemic vascular dysfunction occurs prior to the development of AD, if this occurs in a sex-dependent manner, and whether endothelial cells play a role in the deposition of amyloid beta (Aβ) peptides. We hypothesized that vascular dysfunction occurs prior to the onset of amyloid pathology, thus escalating its progression. Furthermore, endothelial cells from female mice will present with an exacerbated formation of Aβ peptides due to an exacerbated pressure pulsatility. To test this hypothesis, we used a double transgenic mouse model of early-onset AD (APPswe/PSEN1dE9). We evaluated hippocampus-dependent recognition memory and the cardiovascular function by echocardiography and direct measurements of blood pressure through carotid artery catheterization. Vascular function was evaluated in resistance arteries, morphometric parameters in the aortas, and immunofluorescence in the hippocampus and aortas. We observed that endothelial dysfunction occurred prior to the onset of amyloid pathology irrespective of sex. However, during the onset of amyloid pathology, only female APP/PS1 mice had vascular stiffness in the aorta. There was elevated Aβ deposition which colocalized with endothelial cells in the hippocampus from female APP/PS1 mice. Overall, these data showed that vascular abnormalities may be an early marker, and potential mediator of AD, but exacerbated aortic stiffness and pressure pulsatility after the onset of amyloid pathology may be associated with a greater burden of Aβ formation in hippocampal endothelial cells from female but not male APP/PS1 mice.

**Graphical Abstract:**

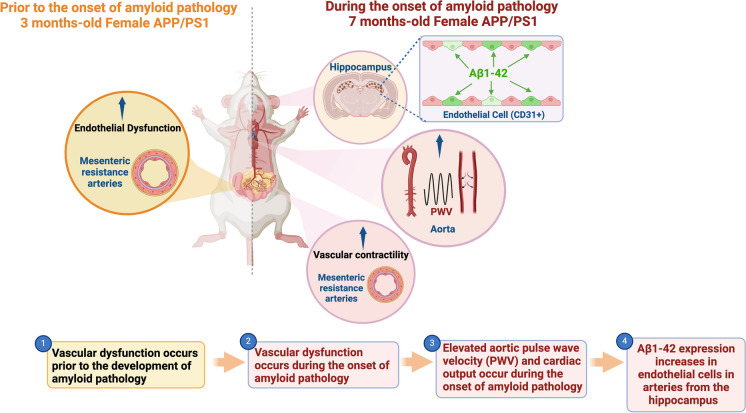

**Supplementary Information:**

The online version contains supplementary material available at 10.1007/s11357-024-01213-0.

## Introduction

Alzheimer’s disease (AD) is the most common form of dementia and according to the US Census and the Chicago Health and Aging Project, 6.7 million Americans aged 65 years and older were living with AD in 2023 [1]. Women are disproportionately affected by AD, comprising two-thirds of all AD patients [1]. However, the cause for this disparity is unknown. The dogma that women tend to live longer than men, and therefore, are more likely to develop AD is not convincing, as the incidence of non-AD dementia is not greater in women.

Alzheimer’s disease was once considered a brain-specific disease, characterized by the increase of neuronal amyloid-β protein (Aβ) and tau neural fibrillary tangles. However, growing evidence supports a robust and likely causal association with cardiovascular diseases, including hypertension, and dementia [2–5]. For instance, a few studies have shown that hypertension-induced lesions and AD may have an additive or synergistic effect and produce a more severe cognitive impairment than either process alone [6, 7]. This premise is corroborated by the Systolic Blood Pressure Intervention Trial (SPRINT) MIND study which observed that intensive anti-hypertensive treatment was associated with a significant reduction in cognitive impairment [4]. Accordingly, there is a reciprocal relationship between Aβ accumulation and cerebrovascular insult, such that Aβ peptide deposition provokes vascular changes similar to those observed in hypertension and diabetes [2, 8]. Vice versa, pathological alterations to the cerebral microvessels in hypertension and impaired Aβ clearance in diabetes can increase Aβ peptides accumulation thus enhancing the pathophysiology of AD [2]. Aβ peptides are toxic to the brain and the peripheral vascular system and can cause cellular damage, enhanced vasoconstriction, and impaired endothelium-dependent vasodilation, hence promoting vascular disease [9]. However, it is unknown whether vascular dysfunction occurs prior to the development of AD, whether this occurs in a sex-dependent manner, and whether vascular tissue could play a role in the deposition of Aβ peptides. To address these questions, we hypothesized that vascular dysfunction occurs prior to the onset of amyloid pathology, which in turn escalates its progression. Furthermore, endothelial cells from female mice will present with an exacerbated formation of Aβ peptides due to an exacerbated pressure pulsatility. We used the double transgenic APPswe/PSEN1dE9 mice, a mouse model of early-onset AD (EOAD). These mice show the onset of amyloid pathology in the form of detectable Aβ plaque deposits by 6–7 months of age. By 9 months, they have numerous plaques comprising of aggregated and diffuse Aβ peptides in the neocortex, hippocampus, thalamus, and cerebellum [10, 11]. The main isoforms of the neurotoxic Aβ peptides include Aβ_1-40_ and Aβ_1-42_, and they are byproducts of the metabolism of the parental amyloid precursor protein (APP) after its sequential proteolytic cleavage by β-secretase (BACE-1) and γ-secretase (PSEN-1 and PSEN-2) [12]. We used male and female mice prior to the onset of amyloid pathology (3 months old; referred to as 3-month-old APP/PS1 mice) or during the onset of amyloid pathology (7 months old; referred to as 7-month-old APP/PS1 mice). Here, we found that vascular dysfunction occurs prior to the onset of amyloid pathology, and Aβ plaque deposits colocalize with endothelial cells in the hippocampus of the female but not male APP/PS1 mice. To the best of our knowledge, this is the first report showing that mesenteric resistance arteries (MRA) exhibit endothelial dysfunction associated with hypocontractility prior to the development of amyloid pathology in a model of early-onset AD. This suggests that more attention from pre-clinical and clinical studies needs to be given to the peripheral vasculature where any interference in the hemodynamics, blood nutrients, and oxygen supply would also affect the brain.

## Materials and methods

### Animals

The mouse strain B6C3Tg (APPswe/PSEN1dE9) 85Dbo/Mmjax (RRID:MMRRC_034832-JAX) was obtained from the Mutant Mouse Resource and Research Center (MMRRC) at The Jackson Laboratory, an NIH-funded strain repository. This strain was originally donated to the MMRRC by David Borchelt, Ph.D., McKnight Brain Institute, University of Florida. Male and female mice were obtained in two age cohorts: prior to the onset of amyloid pathology (3 months old) and during the onset of amyloid pathology (7 months old). The controls were age-matched C57BL/6 J mice (RRID: IMSR_JAX:000664) also obtained from The Jackson Laboratory. All animal procedures and protocols used were approved by the Institutional Animal Care and Use Committee at the University of South Carolina School of Medicine, Columbia, SC (#101,768). Experiments were conducted following the National Institutes of Health Guide for the Care and Use of Laboratory Animals and Animal Research Reporting of in Vivo Experiments (ARRIVE) guidelines. Mice were maintained on a 12-h light/dark cycle with water and standard chow diet ad libitum. Please note that the sample size indicated per experiment is the number of independent mice used. For vascular function experiments, mice were euthanized by thoracotomy and exsanguination via cardiac puncture while anesthetized under isoflurane gas (5% in 100% O_2_). The mesenteric bed was harvested and maintained in cold Krebs solution (please see the complete Krebs solution formulation in the following sections).

### Survival

Starting from the moment the mice arrived at the vivarium, mortality was reported in the early-onset AD mice and the age-matched controls. Age at time of death (in weeks) was recorded and the probability of survival was calculated.

### Novel object recognition (NOR) test

Novel object recognition (NOR) test evaluates the level of object memory recognition in rodents. The test was performed in an open field arena (50 cm × 50 cm) and following a previously published protocol [13, 14]. Lighting conditions were 6–8 lux in the center of the arena throughout the test. The mice were allowed to habituate to the arena for 10 min. After habituation, the mice were placed in the arena with two identical objects. The objects (two unpainted wooden blocks) were placed 3 cm from two opposing walls. The mice were allowed to explore the arena for 10 min as part of the training session. To test for recognition, the mice were immediately placed in the arena for a testing session of 10 min. In the testing session, one of the identical objects from the training session was replaced with a novel object (upside down cup). The time spent investigating each object was recorded. Object recognition was defined as animals spending more time investigating the novel object compared to the familiar one. Locomotor activity and velocity were recorded to control for any locomotor differences. The test was recorded from an overhead camera and animal movement tracked using Ethovision XT automated tracking software (Noldus). Analyses of behaviors were performed by an experimenter blind to the groups.

### Immunofluorescence of the brain hippocampus and aortas

Upon collection, the brains were immersed in 4% paraformaldehyde (PFA) for 24 h. Brains were transferred to 1 × PBS containing 15% sucrose until they sunk, and then in 1 × PBS containing 30% sucrose at 4 °C until they were sectioned. The samples were immersed in Tissue Tek® optimum cutting temperature (O.C.T.) Compound (Sakura, # 4583) and frozen in liquid nitrogen. Coronal sections of the hippocampus at 8 µm thickness were prepared at − 20 °C using a Thermo Scientific Cryostat Microm HM525 (Thermo Scientific). The slices were rinsed in warm 1 × PBS at room temperature for 5 min and then fixed using 4% PFA for 20 min. They were then rinsed twice using warm 1 × PBS and permeabilized at room temperature for 1 h in 1 × PBS and 0.3% triton X-100 with 10% goat serum (50062Z; Invitrogen) and 1% bovine serum albumin (BSA). After, samples were incubated overnight at 4 °C with the following primary antibodies: mouse anti-beta amyloid (1:100; NBP2-13,075, Novus) and mouse/rat CD31/PECAM-1 Alexa Fluor 488-conjugated antibody (1:300; FAB3682G, R&D systems) in 1% BSA. After, they were rinsed twice with warm 1 × PBS and incubated for 1 h at room temperature with the secondary antibody Alexa 647-labeled goat anti-mouse IgG (1:500; 405,322, Biolegend) in 1% BSA. The hippocampus sections were rinsed twice with 1 × PBS, and the coverslips mounted using Fluoroshield™ with DAPI (Sigma Aldrich, F6057). Negative control represented hippocampus without primary antibody incubation.

High-resolution images were acquired on the confocal microscope (Stellaris 5 LIAchroic Confocal System, Leica) 40 × objective.

Aortas isolated from male and female mice were embedded in Tissue Tek® O.C.T. Compound (Sakura, # 4583), frozen in liquid nitrogen, and sectioned (5 µm) using Thermo Scientific Cryostat Microm HM525 (Thermo Scientific). The sections were rinsed in warm 1 × PBS at room temperature for 5 min and then fixed with 4% PFA for 20 min, and permeabilized with 0.1% Triton X-100 and 0.01 M glycine for 1 h at 37 °C. Following, the slides were incubated with 5% BSA for 15 min and subsequently incubated with 5% goat serum for 30 min at room temperature to reduce non-specific binding. After, samples were incubated overnight at 4 °C with the following primary antibodies: mouse anti-beta amyloid (1:100; NBP2-13,075, Novus) and mouse/rat CD31/PECAM-1 Alexa Fluor 488-conjugated antibody (1:300; FAB3682G, R&D systems) in 1% BSA. After, they were rinsed twice with warm 1 × PBS and incubated for 1 h at room temperature with the secondary antibody Alexa 647-labeled goat anti-mouse IgG (1:500; 405,322, Biolegend) in 1% BSA. The aorta sections were rinsed twice with 1 × PBS, and the coverslips mounted using Fluoroshield™ with DAPI (Sigma Aldrich, F6057). Negative control represented aortas with no primary antibody incubation. High-resolution images were acquired on the confocal microscope (Stellaris 5 LIAchroic Confocal System, Leica) 63 × objective.

The LAS-X software was used with the following configurations: 1024 × 1024, 400 Hz, 16 bits, *xyz* on sequential mode, and LAS-X or FIJI software was used for image analysis. In the hippocampus, data were presented as % area (290.62 µm^2^). Colocalization between Aβ_1-42_ and CD31 in the hippocampus was analyzed using LAS-X software and the positive colocalization signal shown in white in the images on the lower panel. Representative orthogonal projections of the *xz* and *yz* axes of the colocalization images with Aβ_1-42_ and endothelial cells were presented in the third and lowest panel. In the aortas, quantification was done on the endothelium using % area (184.52 µm^2^) and in the vascular smooth muscle layer using 10 regions of interest (70 width × 72 height). No measurements were done in the adventitia or perivascular adipose tissue (PVAT).

### Vascular function

Second- and third-order mesenteric resistance arteries (MRA) (with an inner diameter < 200 µm) were isolated and 2 mm length segments mounted on DMT wire myographs (Danish MyoTech, Aarhus, Denmark). The MRA were oxygenated, heated (37 °C), and submerged in Krebs solution in mM (NaCl 130; KCl 4.7; NaHCO_3_ 14.9; CaCl_2_.2H_2_O 1.56; KH_2_PO_4_ 1.18; MgSO_4_.7H_2_O 1.18; EDTA 0.026; glucose 5.6, pH 7.40) to mimic an environment for optimal function. The MRA were normalized to their optimal lumen diameter for active tension development, as described previously by our group [15]. To test vascular smooth muscle integrity, the arteries were initially contracted with 120 mM potassium chloride (KCl) Krebs solution with the following components in mM (NaCl 14.7; KCl 120; NaHCO_3_ 14.9; CaCl_2_.2H_2_O 1.56; KH_2_PO_4_ 1.18; MgSO_4_.7H_2_O 1.18; EDTA 0.026; glucose 5.6, pH 7.40). Cumulative concentration-effect curves to acetylcholine (ACh, 1 pM to 30 µM) following contraction to the selective thromboxane A_2_ receptor agonist, U46619 (30 nM), were performed to evaluate relaxation. Relaxation responses to ACh are shown as a percent of the initial U46619 contraction (30 nM). Cumulative concentration-effect curves to phenylephrine (PE; 0.1 nM to 30 µM) or to U46619 (1 pM to 1 µM) were also performed (tension, mN/mm).

### Cardiac function and vascular stiffness analysis

The electrocardiogram (ECG) and pulse wave velocity (PWV) were performed using the Visual Sonics VEVO 3100 High Resolution In Vivo Imaging System, and data were calculated with the Vevo Lab software. Briefly, anesthesia was induced by putting the mouse in an induction chamber using 3% isoflurane and 2 L/min 100% oxygen for 2 min. Once the animal lost its righting reflex, it was laid supine on a heated platform with its nose enveloped in a nosecone to keep the mouse anesthetized with 1.5% isoflurane [16]. The mouse limbs were taped to four ECG electrodes which were embedded in the platform for the measurement of heart rate, ECG, and respiratory rate. Mouse fur of the anterior chest and abdomen was removed using a depilatory cream. Transthoracic echocardiograph images were obtained by placement of echocardiograph probe in the gel and moving throughout the thoracic region. From a transthoracic approach, 2-dimensional targeted M-mode echocardiographic recordings were obtained in the parasternal short and long axis views. Two-dimensional M-mode echocardiographic recordings were obtained using a 40-MHz scanning head with a spatial resolution of 30 µm. Echocardiographic measurements were made as an average of at least five beats for each measurement. Left ventricle (LV) dimension and wall thickness were made at end-systole and end-diastole using the American Society of Echocardiography and established criteria for mice. Mean wall thickness was calculated as the average of the end diastolic left ventricular anterior wall thickness (LVAW d) and left ventricular posterior wall thickness (LVPW d). LV mass was calculated using standard formula on VEVO Lab software. Left ventricle end diastolic volume and end systolic volume were determined using Simpson’s method of disks and used to compute ejection fraction (EF). The ascending and descending aortic peak velocities were measured from the pulse wave (PW) Doppler-mode aortic arch view. Pulse wave velocity was obtained from the B-mode and Doppler-mode aortic arch view, calculated as PWV = aortic arch distance/transit time (cm·s^−1^) [17]. The PW Doppler mode sample volume was placed in the ascending aorta and the time (T1) from the onset of the QRS complex to the onset of the ascending aortic Doppler waveform was measured. On the same image plane, the PW Doppler mode sample volume was placed as distal as possible in the descending aorta and the time (T2) from the onset of the QRS complex to the onset of the descending aortic Doppler waveform was measured. Obtained values for T1 and T2 were averaged over 5 cardiac cycles. The aortic arch distance was measured between the 2 sample volume positions along the central axis of aortic arch on the B-mode image, and the transit time was calculated by T2 − T1 (ms) [17].

### Blood pressure measurement by left carotid catheterization

The blood pressure was measured using a procedure described by Zhao et al. [18]. The mice were anesthetized with 3% isoflurane and 2 L/min 100% oxygen and placed in the supine position on a warm pad. An incision in the skin (1 cm) was made on the left side of the neck, near the mouse trachea, and the left carotid artery exposed and carefully separated from other neighboring structures including the vagus nerve. A silk suture was placed distally (closer to the head) for the complete ligation of the vessel. A second silk suture was placed proximally (closer to the heart) to allow temporary obstruction of blood flow. Finally, a third silk suture was placed loosely between the first two ligatures and a small incision (arteriotomy) was made between the first and the third suture. The tip of a catheter that had been pre-filled with 2% heparinized sterile saline was inserted into the carotid artery via the arteriotomy in the direction of the heart and secured in place by tying the third suture once the catheter had been advanced past the ligature. The isoflurane anesthesia was reduced to 1%, and the pulsatile arterial pressure (PAP, in mmHg) was acquired for 20 min after stabilization of signal, using the LabChart 7 software. The systolic blood pressure (SBP, in mmHg) was recorded and analyzed using the LabChart 7 software formulas. Pressure calibration was performed prior to the experimentation protocol. The SBP was analyzed as the mean of 10 different stable maximum values taken in 1-min intervals and plotted on GraphPad Prism 10.1.2 as the mean ± standard error mean (S.E.M.).

After blood pressure measurements, blood was collected through the arterial catheter in chilled heparinized tubes under anesthesia (5% isoflurane). Plasma was obtained after centrifugation at 1000 × g for 15 min at 4 °C and stored at − 80 °C until further experiments.

### Plasma Aβ_1-40_ levels

The level of Aβ_1-40_ in plasma samples was determined using a commercial ELISA kit (Elabscience, E-EL-M3009) according to the manufacturer’s instructions. Briefly, plasma samples were obtained from 3- and 7-month-old male and female APP/PS1 and control mice. A total volume of 100 µL of the standard or sample were added to each well and incubated for 90 min at 37 °C. The samples were incubated with 100 µL of the biotinylated detection Ab/Ag for 1 h at 37 °C. Then, the samples were washed three times and incubated with 100 µL of HRP conjugate for 30 min at 37 °C. Substrate reagent (90 µL) was added and incubated for 15 min at 37 °C. Finally, 50 µL of stop solution was APP/PS1ded and the optical density (OD) determined using a spectrophotometer at 450 nm and the data presented as pg/mL. Samples were evaluated in duplicate from 4 different animals and processed on the same day.

### Second harmonic generation (SHG)

Mesenteric resistance arteries isolated from male and female control and APP/PS1 mice were investigated for their collagen and elastin content by second harmonic generation (SHG) signal using multiphoton microscopy. Briefly, MRA were isolated from mice, cleaned of adjacent tissue, embedded in Tissue-Tek® O.C.T. Compound (Optimal Cutting Tissue; Sakura, #4583), frozen in dry ice, and stored at − 80 °C until experiments. Following, sample Sections (5 µm) were incubated with PBS for 5 min at room temperature, and Fluoroshield™ with DAPI (Sigma Aldrich, F6057) was used as mounting media and for nuclei staining. Collagen, elastin, and nuclei were visualized using Leica SP8 multiphoton MP system (Leica Microsystems, Mannheim, Germany), with pulsed femtosecond titanium:sapphire (Ti:Sa) laser (Chameleon Vision II, Coherent, Santa Clara, CA, USA) tunable for 880 nm (for collagen) and 720 nm (for DAPI). Multiphoton excitation at 880 nm was used to reveal structural information of collagen fibers by intrinsic contrast imaging of the SHG signal. Elastin autofluorescence was observed at 488 nm (498 to 550 nm). Images were acquired on a 25 × water objective, using *xyz* series on a sequential mode with high resolution (1024 × 1024 format, 600 Hz, 16 bits), and 3D reconstructions were automatically performed with LAS-X software. Images were analyzed by FIJI software using max projections of *z* series, and percentage (%) of collagen area or elastin area was analyzed. The total area corresponded to 442.86 µm^2^ at 25 × objective.

### Western blot for measurement of β-secretase (BACE-1) and presenilin-1 (*PSEN*-1) expression

Protein expression of BACE-1 and PSEN-1 in thoracic aortas isolated from male and female APP/PS1 and control mice were evaluated by western blotting. Briefly, frozen aortas were homogenized in lysis RIPA buffer containing protease and phosphatase inhibitors. Thirty micrograms of protein was loaded in 12% SDS-PAGE, and transferred to a nitrocellulose membrane, followed by blocking with 5% milk solution for 2 h, and overnight incubation with the primary antibodies at 4 °C: rabbit monoclonal anti-presenilin 1 (1:1000; Abcam, ab76083; observed band at 18 kDa) or rabbit monoclonal anti-BACE 1 antibody (1:1000; Abcam, AB183612, observed bands between 68 and 70 kDa). Secondary antibodies donkey anti-rabbit HRP (1:1000; Jackson Immuno Research #711–035-152) were incubated for 1 h at room temperature. Chemiluminescence was detected using Pierce™ ECL western blotting substrate (Thermo Fisher Scientific, #32,106). Ponceau red staining was used to check protein transfer after electrophoresis and to normalize the target bands. Data were analyzed by ImageJ software (National Institutes of Health, NIH) and plotted on GraphPad Prism 10.1.2.

### Morphometric analysis of aortas

To analyze arterial remodeling, thoracic mouse aortas were fixed in 10% phosphate-buffered formaldehyde (pH 7.0) for 24 h, dehydrated in 70% ethanol, embedded in paraffin, cut at 5 µm, and then stained with hematoxylin and eosin to analyze the wall thickness, cross-section area (CSA), and wall/lumen (W/L) ratio. The aortic wall thickness was calculated as (external diameter − internal diameter)/2*1000 (µm). Wall-to-lumen ratio (W/L) was calculated by wall thickness/lumen diameter. The CSA was calculated as (external diameter^2^ − internal diameter^2^)*(3.14/4)*1000 (µm). Images were captured using the Invitrogen EVOS FL Auto Cell Imaging System at 10 × and 40 × and analyzed by ImageJ.

### Plasma estradiol levels

The estrogen levels in plasma samples from 3- and 7-month-old female APP/PS1 and control mice were determined using a commercial ELISA kit (Cayman Chemical, #501,890). Briefly, the assay-specific reagents included estradiol acetylcholinesterase (AChE) tracer and estradiol ELISA antiserum. Fifty microliters of samples and standard solutions were transferred to their respective wells. Subsequently, 50 µL of estradiol AChE tracer and 50 µL of estradiol antiserum were added to their designated wells, mixed gently, and then incubated for 120 min at room temperature. Afterward, the liquid was discarded, and the wells were washed four times with a washing buffer. Following the washing process, 200 µL of Elman’s work reagent was added and incubated for 60 min at room temperature in the dark. At the end, the absorbance of the resulting reagent was measured at 405 nm, and data were presented as pg/mL. Samples were evaluated in duplicate from 5 to 6 different animals processed on the same day.

### Statistical analysis

All statistical analyses were performed using GraphPad Prism 10.1.2 (GraphPad Software Inc., La Jolla, CA, USA). Data are presented as mean ± standard error mean (S.E.M.) and statistical significance was set at *p* < 0.05. The pharmacological parameters of agonist maximum effect (Emax, in %) were calculated for each agonist using the non-linear curve regression of the specific cumulative concentration-effect curves. The unpaired and two-tailed Student’s *t*-test (*p* < 0.05) was used to compare two different groups, or the two-way ANOVA, followed by Tukey post hoc (*p* < 0.05), was used to identify interaction factors and compare 3 or more groups. For the survival curve, we used the Mantel-Cox test. The sample size (*n*) indicated per experiment is the number of independent mice used.

## Results

### Female APP/PS1 mice exhibit greater mortality compared to control mice

**Fig. 1:**
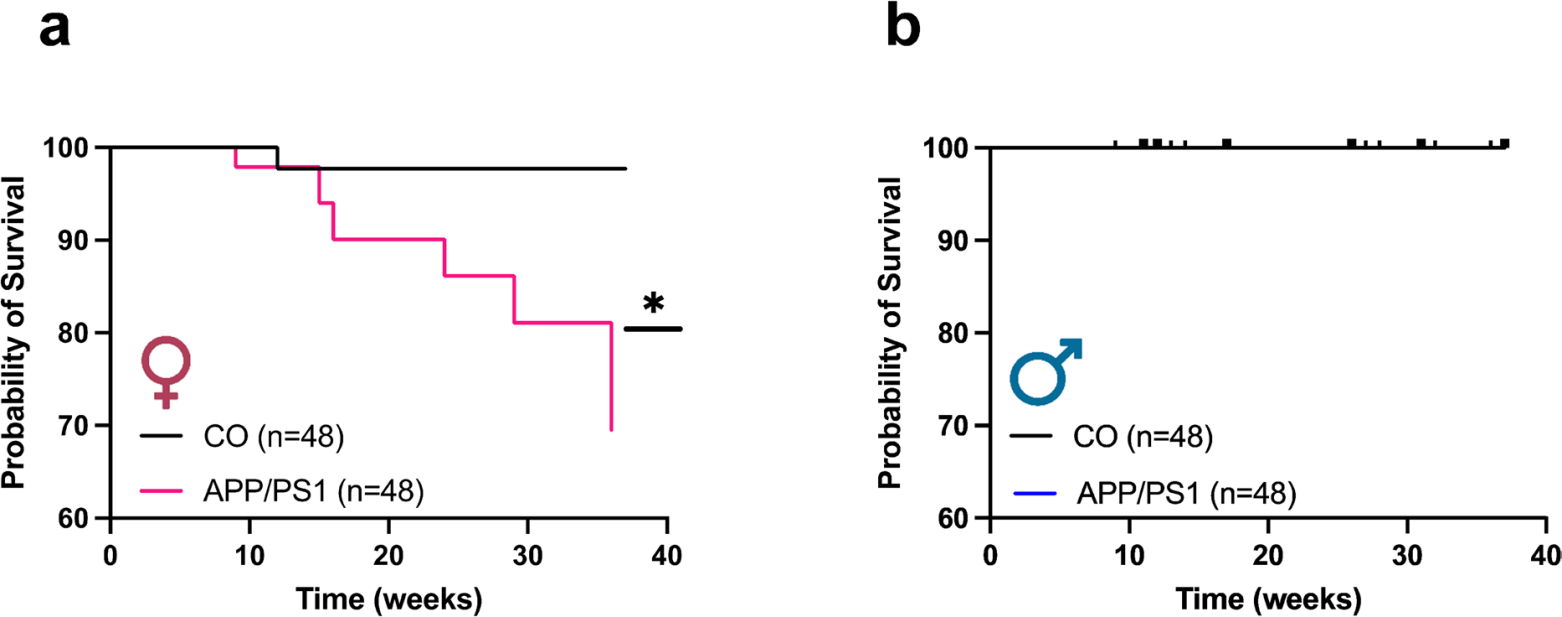
Female APP/PS1 mice exhibit higher mortality. Survival curve analysis for female (**a**) and male (**b**) APP/PS1 and C57BL/6 J mice. Abbreviations: APP/PS1, Alzheimer’s disease; CO, control. The curve was analyzed using the log-rank (Mantel-Cox) test. *Different from female CO (*p* < 0.05) (female CO *n* = 48, APP/PS1 *n* = 48) (male CO *n* = 48, APP/PS1 *n* = 48)

We observed a striking difference in the survival rates of female APP/PS1 mice compared to the age-matched controls. These animals exhibited mortality starting from 9 weeks of age (Fig. 1a). On the other hand, we did not observe any mortality among the male mice (Fig. 1b).

### Cognitive decline occurs in male mice during the onset of amyloid pathology

**Fig. 2:**
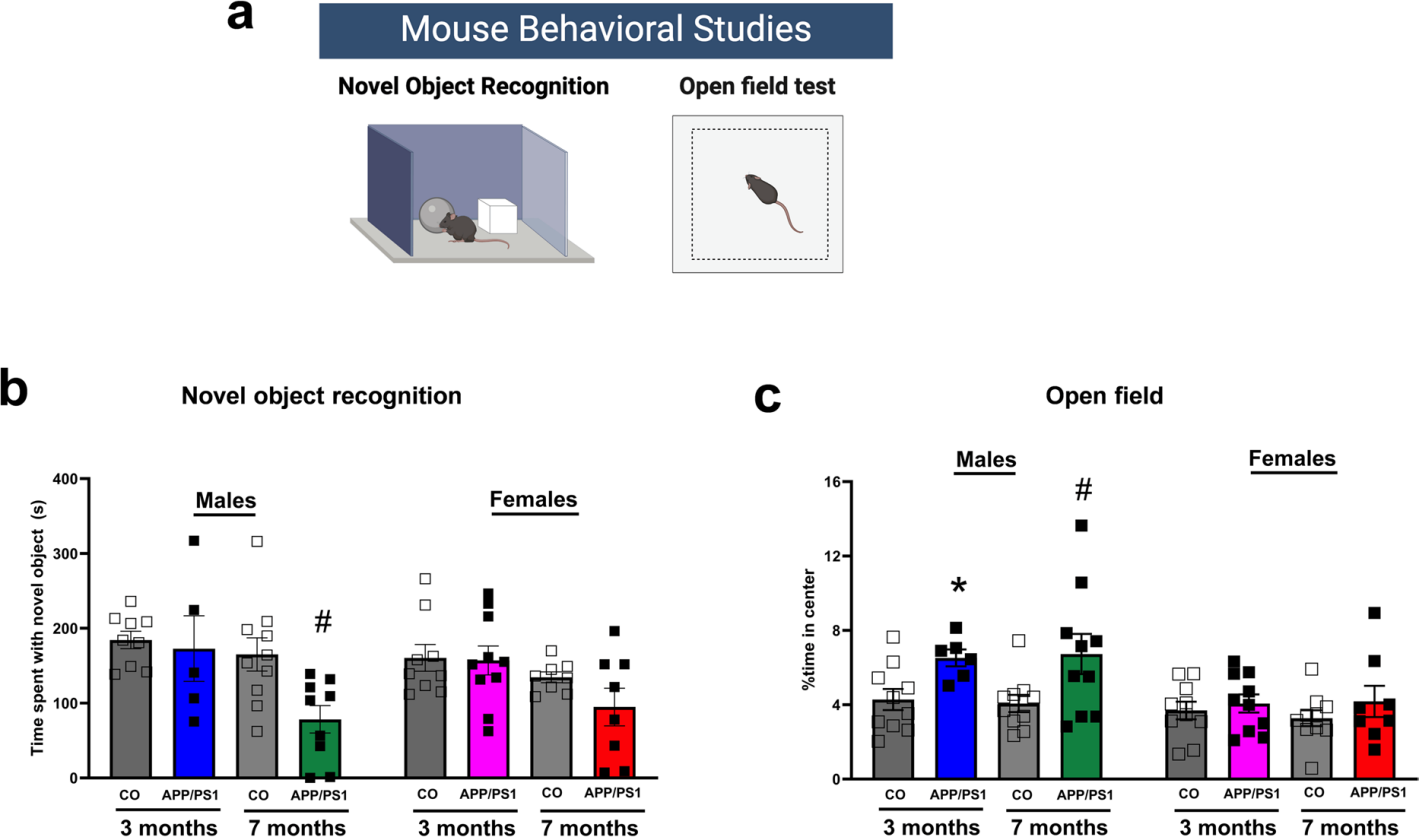
Male APP/PS1 mice present with cognitive decline during the onset of amyloid pathology. Graphical illustration demonstrates the novel object recognition (NOR) and open field tests performed to evaluate mouse behavior (**a**). NOR test in male and female APP/PS1 or C57BL/6 J mice prior to (3 months old) or during the onset of amyloid pathology (7 months old) (male 3 month CO *n* = 9, APP/PS1 *n* = 5; male 7 month CO *n* = 10, APP/PS1 *n* = 9) (female 3 month CO *n* = 9, APP/PS1 *n* = 10; female 7 months CO *n* = 8, APP/PS1 *n* = 8) (**b**). Open field test in male and female APP/PS1 or C57BL/6 J mice prior to (3 months old) or during the onset of amyloid pathology (7 months old) (male 3 month CO *n* = 10, APP/PS1 *n* = 6; male 7 month CO *n* = 10, APP/PS1 *n* = 10) (female 3 month CO *n* = 9, APP/PS1 *n* = 10; female 7 months CO *n* = 9, APP/PS1 *n* = 8) (**c**). Graphs show the time spent with the novel object and the % time spent in the center for the open field respectively. Abbreviations: APP/PS1, Alzheimer’s disease; CO, control. Data are presented as mean ± S.E.M. *Different from 3 months old male CO, # different from 7 months old male CO, two-way ANOVA, Sidak post hoc test (*p* < 0.05). Graphical illustration was created with BioRender.com

To evaluate the cognitive decline in APP/PS1 mice, we performed the NOR test. Figure 2a is a graphical illustration demonstrating the novel object recognition (NOR) and open field tests performed to evaluate mouse behavior. As shown in Fig. 2b, we observed that APP/PS1 mice at the age of 3 months spent similar durations with the novel object as the control mice irrespective of sex. However, the 7-month-old male APP/PS1 mice showed a significant decrease in the time spent with the novel object compared to the controls, suggesting poor recognition in the male mice group during the onset of amyloid pathology. We did not observe any differences in locomotion, as neither the distance traveled (Online Resource 1a) nor the velocity (Online Resource 1b) was different in the NOR test regardless of age or sex. In addition, the male APP/PS1 mice at 3 and 7 months of age exhibited decreased avoidance behavior in the open field compared to the controls, but the female APP/PS1 mice did not show similar effects regardless of strain or age (Fig. 2c). This shows that the observed effects in NOR are not due to potential avoidance behavior related to the open field in which the NOR was performed, as both 3- and 7-month-old male APP/PS1 mice exhibited decreased avoidance behavior in the open field during habituation. The 7-month-old male and female APP/PS1 mice had increased open field distance traveled (Online Resource 1c). However, no differences were observed in the open field velocity (Online Resource 1d) regardless of age and sex, and there were no significant differences between groups in the total time spent exploring (Online Resource 2e).

### Increased Aβ_1-42_ deposits colocalize with endothelial cells in the hippocampus from female mice during the onset of amyloid pathology

**Fig. 3:**
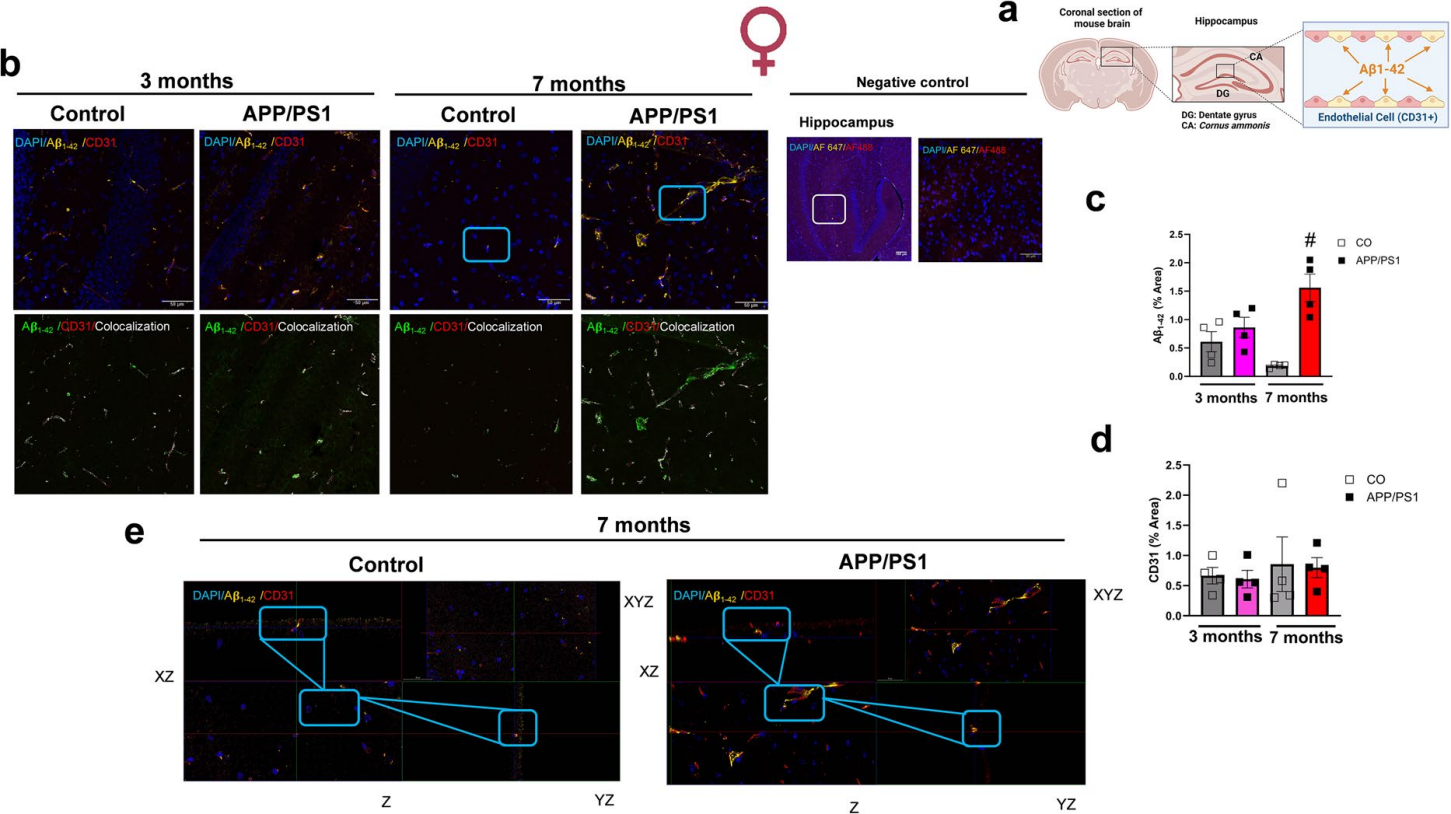
Female APP/PS1 mice have increased hippocampal Aβ_1-42_ plaque deposits which colocalize with endothelial cells. Graphical illustration shows coronal section of the brain and the hippocampus (**a**). Representative images of mice hippocampus showing Aβ_1-42_ plaque burden. Upper panels are merged images showing DAPI (blue; nuclei), Aβ_1-42_ (yellow), and CD31 (red) in the hippocampus of female APP/PS1 or C57BL/6 J mice prior to (3 months old) or during the onset of amyloid pathology (7 months old). Bar represents 50 µm (40 × objective, 290.62 µm^2^ area). Negative controls were the hippocampus with secondary antibody (Alexa Fluor 647 or Alexa Fluor 488) and DAPI. Selected region of interest (white box) shows higher magnification of the hippocampus (40 × objective). Lower panel, colocalization images showing Aβ_1-42_ (in green) and CD31 (in red) (**b**). Graphs show Aβ_1-42_ quantification (% area) (female 3 month CO *n* = 4, APP/PS1 *n* = 4; female 7 months CO *n* = 4, APP/PS1 *n* = 4) (**c**) and CD31 quantification (% area) (female 3 month CO *n* = 4, APP/PS1 *n* = 4; female 7 months CO *n* = 4, APP/PS1 *n* = 4) (**d**). The third and lowest panel shows the *xz* and *yz* axes of the orthogonal projections for the colocalization images between Aβ_1-42 _(yellow) and endothelial cells (red) in the hippocampus from 7-moth-old female C57BL/6J or APP/PS1 mice. Bar represents 50 μm **(e)**. Abbreviations: APP/PS1, Alzheimer’s disease; CO, control. Data are presented as mean ± S.E.M. #Different from 7 months old female CO. Statistics: two-way ANOVA with Tukey post hoc analysis (*p* < 0.05). Graphical illustration was created with BioRender.com

**Fig. 4:**
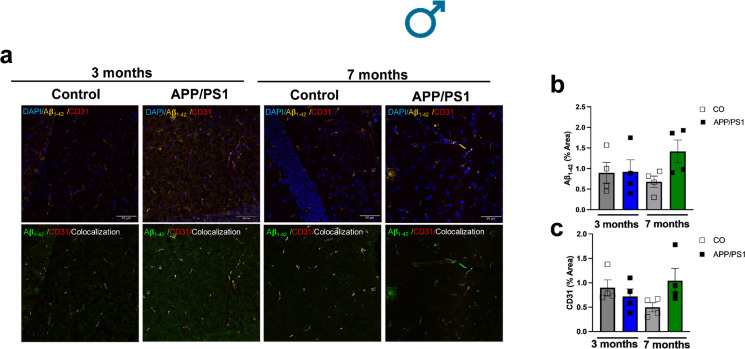
Male APP/PS1 mice did not exhibit significantly increased hippocampal Aβ_1-42_ plaque deposits. (**a)** Representative images of mice hippocampus showing Aβ_1-42_ plaque burden. Upper panels are merged images showing DAPI (blue; nuclei), Aβ_1-42_ (yellow), and CD31 (red) in the hippocampus of male APP/PS1 or C57BL/6 J mice prior to (3 months old) or during the onset of amyloid pathology (7 months old). Bar represents 50 µm (40 × objective, 290.62 µm.^2^ area). Lower panel, colocalization images showing Aβ_1-42_ (in green) and CD31 (in red). Graphs show Aβ_1-42_ quantification (% area) (male 3 month CO *n* = 4, APP/PS1 *n* = 4; male 7 month CO *n* = 4, APP/PS1 *n* = 4) (**b**) and CD31 quantification (% area) (male 3 month CO *n* = 4, APP/PS1 *n* = 4; male 7 month CO *n* = 4, APP/PS1 *n* = 4) (**c**). Abbreviations: APP/PS1, Alzheimer’s disease; CO, control. Data are presented as mean ± S.E.M. Statistics: two-way ANOVA with Tukey post hoc analysis (*p* > 0.05)

The hippocampus which is involved in the spatial and non-spatial recognition memory was stained to evaluate the amount of Aβ_1-42_ plaque in the APP/PS1 and control animals prior to and during the onset of amyloid pathology (Fig. 3a). No differences of the amounts of Aβ deposition were observed between the female APP/PS1 and control mice at the age of 3 months (Fig. 3b). However, Aβ_1-42_ plaque was significantly increased in the 7-month-old female APP/PS1 group compared to the controls (Fig. 3b), confirming that the Aβ deposition is not present at young ages (e.g., 3 months), but increases with age in APP/PS1 mice. Interestingly, we observed that the Aβ deposits colocalize with CD31, which is a marker for vascular endothelial cells (Fig. 3a, c, and d). No differences in this parameter were observed in male mice regardless of strain and age (Fig. 4a–c). These data suggest that vascular endothelial cells could be associated with the deposition of Aβ_1-42 _and play a role in the progression of the AD pathology.

### Endothelial dysfunction occurs prior to the onset of amyloid pathology in isolated MRA

**Fig. 5:**
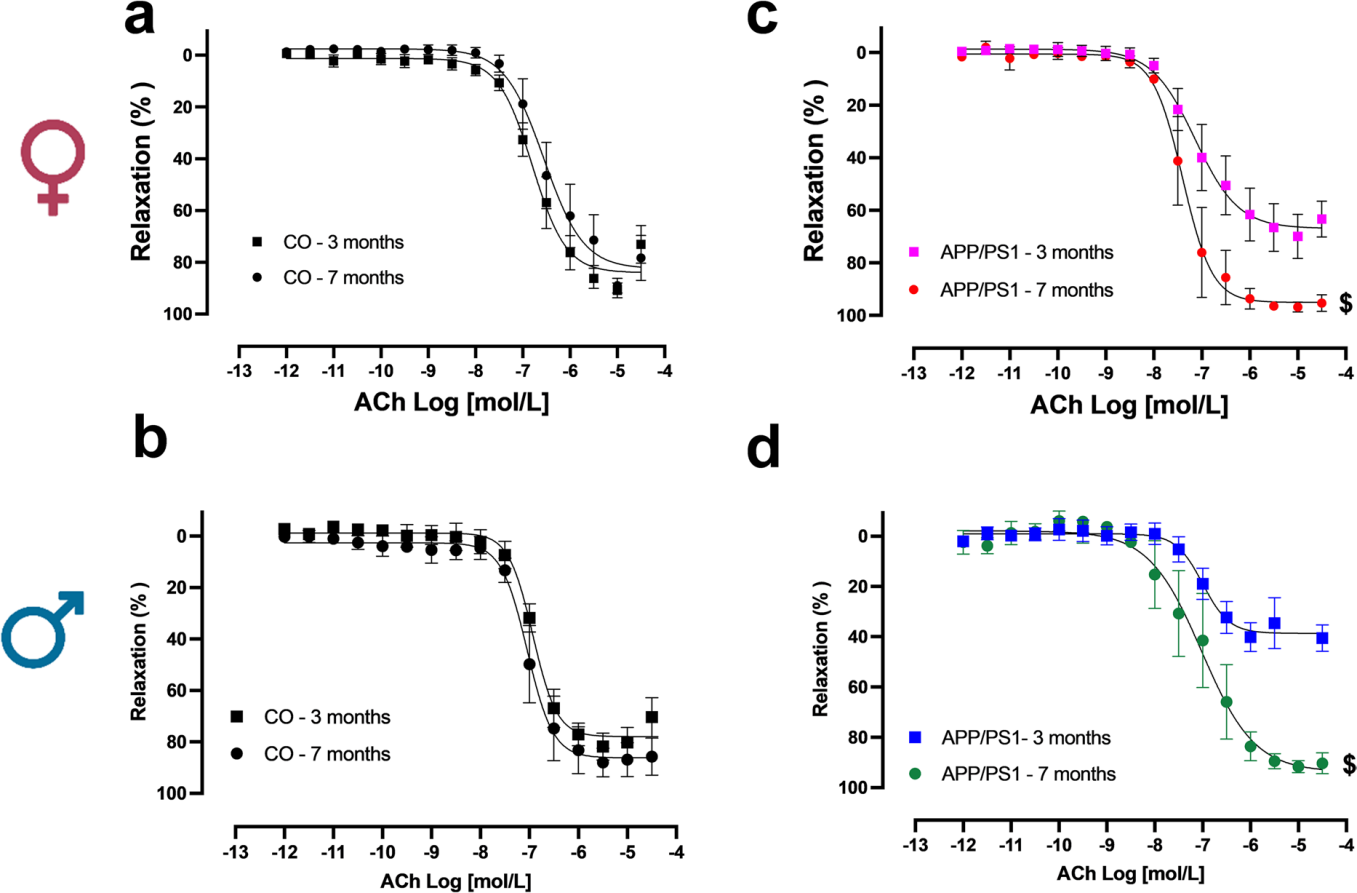
Endothelial dysfunction occurs prior to the onset of amyloid pathology in isolated mesenteric resistance arteries. Cumulative concentration-effect relaxation curves to acetylcholine (ACh) in mesenteric resistance arteries (MRA) isolated from female C57BL/6 J (female 3 month CO *n* = 4, 7 month CO *n* = 6) (**a**) or APP/PS1 mice (female 3 month APP/PS1 *n* = 6, 7 month APP/PS1 *n* = 4) (**c**) and male C57BL/6 J (male 3 month CO *n* = 5, 7 month CO *n* = 3) (**b**) or APP/PS1 mice (male 3 month APP/PS1 *n* = 4, 7 month CO *n* = 5) (**d**) prior to (3 months old) or during the onset of amyloid pathology (7 months old). Abbreviations: APP/PS1, Alzheimer’s disease; CO, control. Data are presented as mean ± S.E.M. $Different from 3 months old female or male APP/PS1. Statistics: Student’s *t*-test (*p* < 0.05)

Cumulative concentration-effect curves to acetylcholine (ACh), a muscarinic receptor agonist, were performed to test the endothelial function on MRA isolated from APP/PS1 mice. While arteries from 3- and 7-month-old female (Fig. 5a) and male (Fig. 5b) control mice showed full relaxation, we observed significant differences in APP/PS1 mice. Interestingly, both female and male APP/PS1 mice (Fig. 5c and d) at 3 months of age showed an evident endothelial dysfunction (reduced relaxation to ACh). However, during the onset of amyloid pathology (7-month-old), both female and male APP/PS1 mice showed a restoration or a “functional catch-up mechanism” of the ACh-induced relaxation (Fig. 5c and d) compared to the 3-month-old mice.

### Isolated MRA from APP/PS1 mice had an exacerbated vascular hypercontractility during the onset of amyloid pathology regardless of sex

**Fig. 6:**
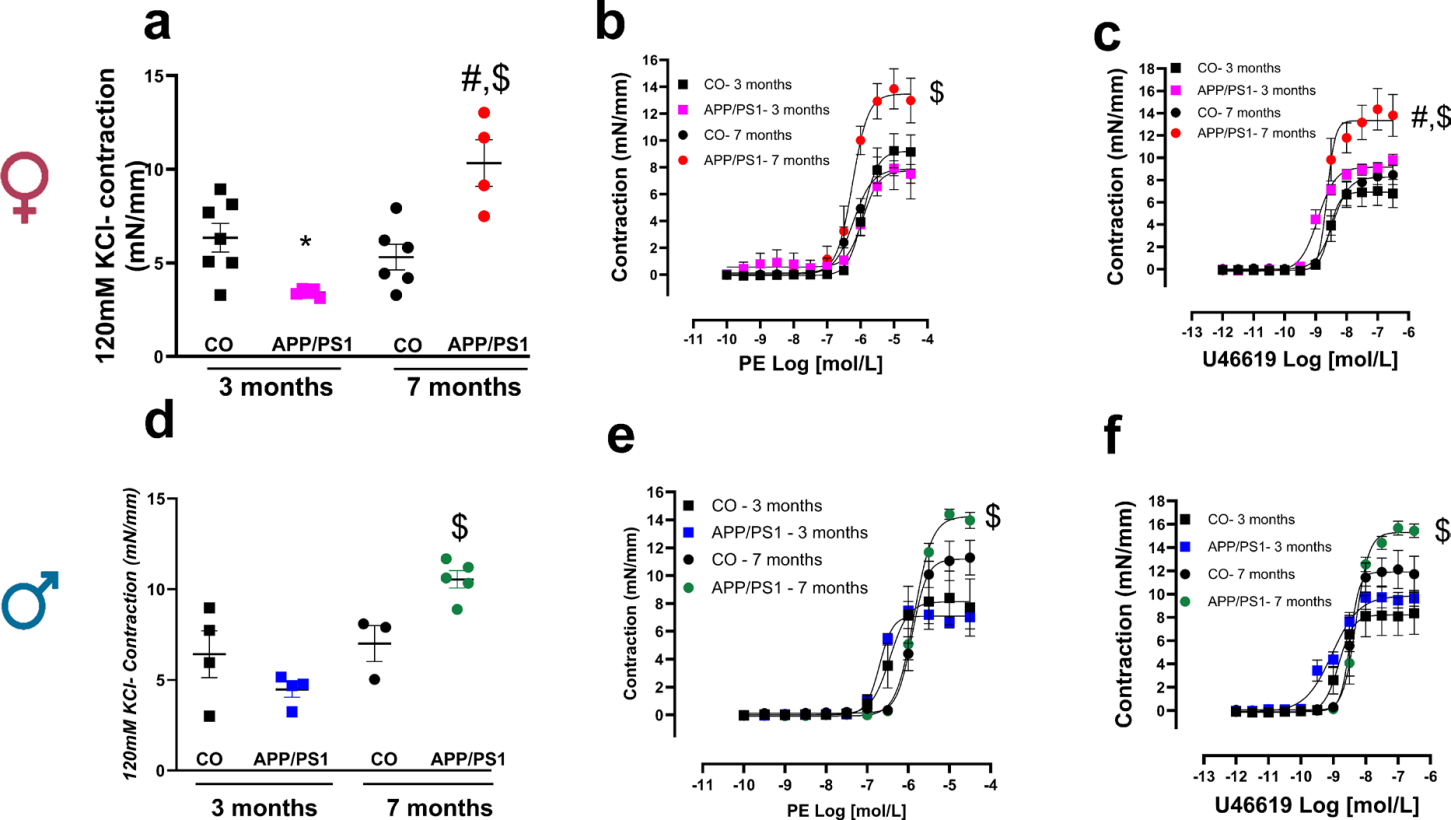
Male and female APP/PS1 mice present with exacerbated vascular hypercontractility during the onset of amyloid pathology. Vascular reactivity assay showing KCl (120 mM)-induced contraction in females (female 3 month CO *n* = 7, APP/PS1 *n* = 5; female 7 months CO *n* = 6, APP/PS1 *n* = 4) (**a**) and males (male 3 month CO *n* = 4, APP/PS1 *n* = 4; male 7 month CO *n* = 3, APP/PS1 *n* = 5) (**d**). Concentration-effect curves to phenylephrine (PE) in females (female 3 month CO *n* = 7, APP/PS1 *n* = 6; female 7 months CO *n* = 6, APP/PS1 *n* = 4) (**b**) and males (male 3 month CO *n* = 5, APP/PS1 *n* = 4; male 7 month CO *n* = 3, APP/PS1 *n* = 5) (**e**) and U46619 in females (female 3 month CO *n* = 4, APP/PS1 *n* = 6; female 7 months CO *n* = 6, APP/PS1 *n* = 4) (**c**) and males (male 3 month CO *n* = 5, APP/PS1 *n* = 4; male 7 month CO *n* = 3, APP/PS1 *n* = 5) (**f**) in mesenteric resistance arteries (MRA) prior to (3 months old) or during the onset of amyloid pathology (7 months old) in APP/PS1 or C57BL/6 J male and female mice. Abbreviations: APP/PS1, Alzheimer’s disease; CO, control. Data are presented as mean ± S.E.M. *Different from 3 months old female CO, #different from 7 months old female CO, $different from 3 months old female or male APP/PS1. Statistics: two-way ANOVA with Tukey post hoc analysis (*p* < 0.05)

In order to determine if changes in vascular contractility could occur prior to or during the onset of amyloid pathology, we performed cumulative concentration-effect curves to three different contractile factors (1) phenylephrine (PE) and (2) U46619 (agonist-dependent contraction) and (3) KCl 120 mM (agonist-independent contraction) in MRA from all groups. Interestingly, arteries from 3-month-old female APP/PS1 mice exhibited a lower contraction induced by the non-selective depolarizing KCl solution compared to the controls (Fig. 6a). Arteries from 3-month-old male APP/PS1 mice also exhibited a tendency toward reduced contraction, although statistical significance was not reached (Fig. 6d). However, arteries from 7-month-old male and female APP/PS1 mice presented hypercontractility to all agonists (PE, KCl, and U46619) compared to the controls (Fig. 6a–f). Interestingly, when the area under the curve (AUC) was evaluated, the 7-month-old female APP/PS1 exhibited greater hypercontractility to PE compared to the male APP/PS1 mice at the same age (PE AUC in 7-month-old female control: 14 ± 2.2; 7-month-old female APP/PS1: 24 ± 2.4; 7-month-old male control: 22 ± 2.4; 7-month-old male APP/PS1: 20 ± 1.1). A similar effect was observed with U46619 (U46619 AUC in 7-month-old female control: 16 ± 1.3; 7-month-old female APP/PS1: 34 ± 4.4; 7-month-old male control: 30 ± 4.6; 7-month-old male APP/PS1: 35 ± 1.2). No differences were observed in the lumen diameter between the groups (in µm: 3-month-old female control: 171 ± 4.7; 3-month-old female APP/PS1: 170 ± 6.7; 7-month-old female control: 197 ± 5.9; 7-month-old female APP/PS1: 171 ± 8.7; 3-month-old male control: 166 ± 5.6; 3-month-old male APP/PS1: 164 ± 7.1; 7-month-old control: 157 ± 2.7 µm; 7-month-old APP/PS1:159 ± 10). Furthermore, there were no significant differences observed in the collagen or elastin from the MRA isolated from control and APP/PS1 mice regardless of age or sex (Online Resource 3a-e).

### Increased aortic stiffness and cardiac output in female APP/PS1 mice and reduced SBP in male APP/PS1 mice during the onset of amyloid pathology

**Fig. 7:**
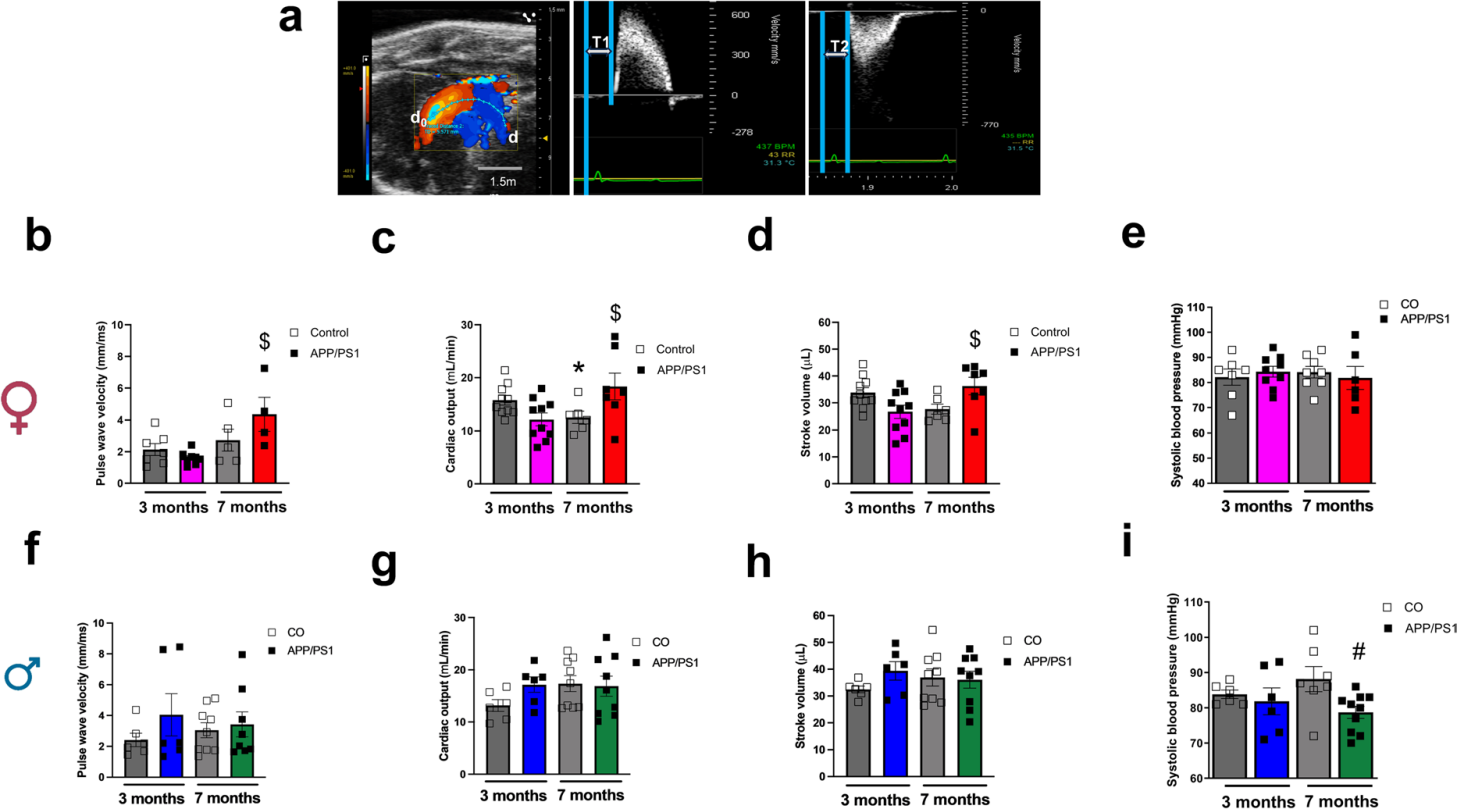
Female APP/PS1 have increased aortic stiffness, cardiac output, and stroke volume and male APP/PS1 mice have lower blood pressure levels during the onset of amyloid pathology. Representative image obtained from the B-mode and Doppler-mode aortic arch view to calculate pulse wave velocity (PWV) **(a)**. The PW Doppler mode sample volume was placed in the ascending aorta and the time (T1) from the onset of the QRS complex to the onset of the ascending aortic Doppler waveform was measured. On the same image plane, the PW Doppler mode sample volume was placed as distal as possible in the descending aorta and the time (T2) from the onset of the QRS complex to the onset of the descending aortic Doppler waveform measured. Pulse wave velocity (PWV) analysis of vascular stiffness in female (female 3 month CO *n* = 8, APP/PS1 *n* = 9; female 7 months CO *n* = 6, APP/PS1 *n* = 4) (**b**) and male (male 3 month CO *n* = 6, APP/PS1 *n* = 6; male 7 month CO *n* = 8, APP/PS1 *n* = 8) (**f**) mice; cardiac output from female (female 3 month CO *n* = 10, APP/PS1 *n* = 10; female 7 months CO *n* = 6, APP/PS1 *n* = 7) (**c**) and male (male 3 month CO *n* = 6, APP/PS1 *n* = 6; male 7 month CO *n* = 9, APP/PS1 *n* = 9) (**g**) mice; stroke volume from female (female 3 month CO *n* = 10, APP/PS1 *n* = 10; female 7 months CO *n* = 6, APP/PS1 *n* = 7) (**d**) and male (male 3 month CO *n* = 6, APP/PS1 *n* = 6; male 7 month CO *n* = 9, APP/PS1 *n* = 9) (**h**) mice; and systolic blood pressure measured by left carotid catheterization in female (female 3 month CO *n* = 7, APP/PS1 *n* = 9; female 7 months *n* = 8, APP/PS1 *n* = 6) (**e**) and male (male 3 month CO *n* = 6, APP/PS1 *n* = 6; male 7 month CO *n* = 7, APP/PS1 *n* = 10) (**i**) APP/PS1 or C57BL/6 J mice prior to (3 months old) or during the onset of amyloid pathology (7 months old). PWV is presented in mm/ms; cardiac output is presented in mL/min; stroke volume is presented as µL; and systolic blood pressure is presented in mmHg. Abbreviations: APP/PS1, Alzheimer’s disease; CO, control. Data are presented as mean ± S.E.M. $Different from 3 months old female APP/PS1, *different from 3 months old female CO, #different from 7 months old male CO. Statistics: two-way ANOVA with Tukey post hoc analysis (*p* < 0.05)

**Fig. 8:**
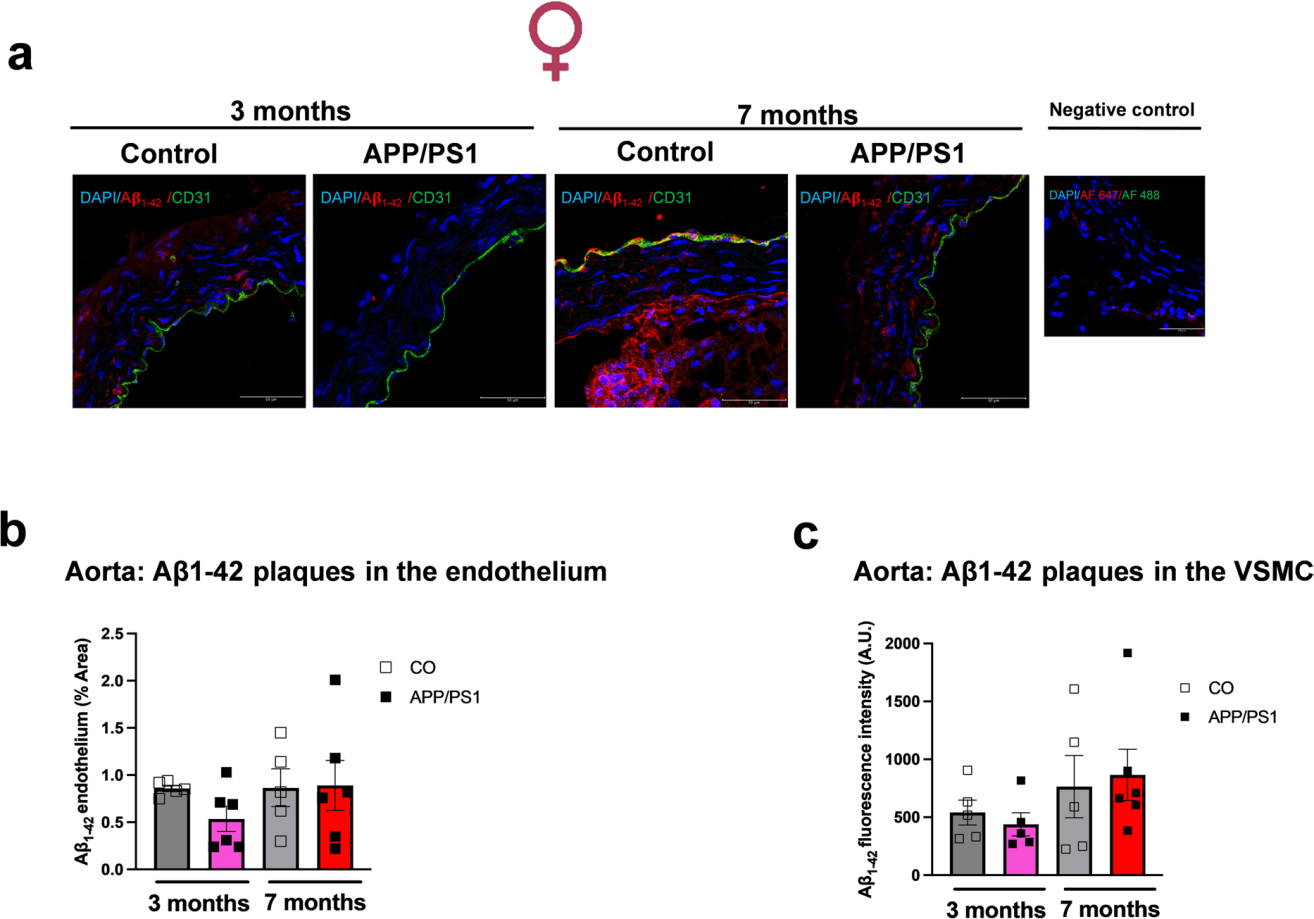
Aorta from female APP/PS1 mice did not have increased Aβ_1-42_ plaque deposition. Representative images of mice aorta showing Aβ_1-42_ plaque burden. Merged images show DAPI (blue; nuclei), Aβ_1-42_ (red), and CD31 (green) in the aortas from female APP/PS1 or C57BL/6 J mice prior to (3 months old) or during the onset of amyloid pathology (7 months old) (**a**). Bar represents 50 µm (63 × objective, 184.52 µm.^2^ area). Negative controls were aortas with secondary antibody (Alexa Fluor 647 or Alexa Fluor 488) and DAPI. Graphs show the Aβ_1-42_ quantification (% area) in the endothelium (**b**) and the vascular smooth muscle cells (VSMC, **c**). The VSMC were quantified through 10 regions of interest (70 × 72) and presented as Aβ_1-42_ fluorescence intensity (A.U.). Abbreviations: APP/PS1, Alzheimer’s disease; CO, control. Data are presented as mean ± S.E.M. Statistics: two-way ANOVA with Tukey post hoc analysis (*p* > 0.05) (female 3 month CO *n* = 5, APP/PS1 *n* = 6; female 7 months CO *n* = 5, APP/PS1 *n* = 6)

**Fig. 9:**
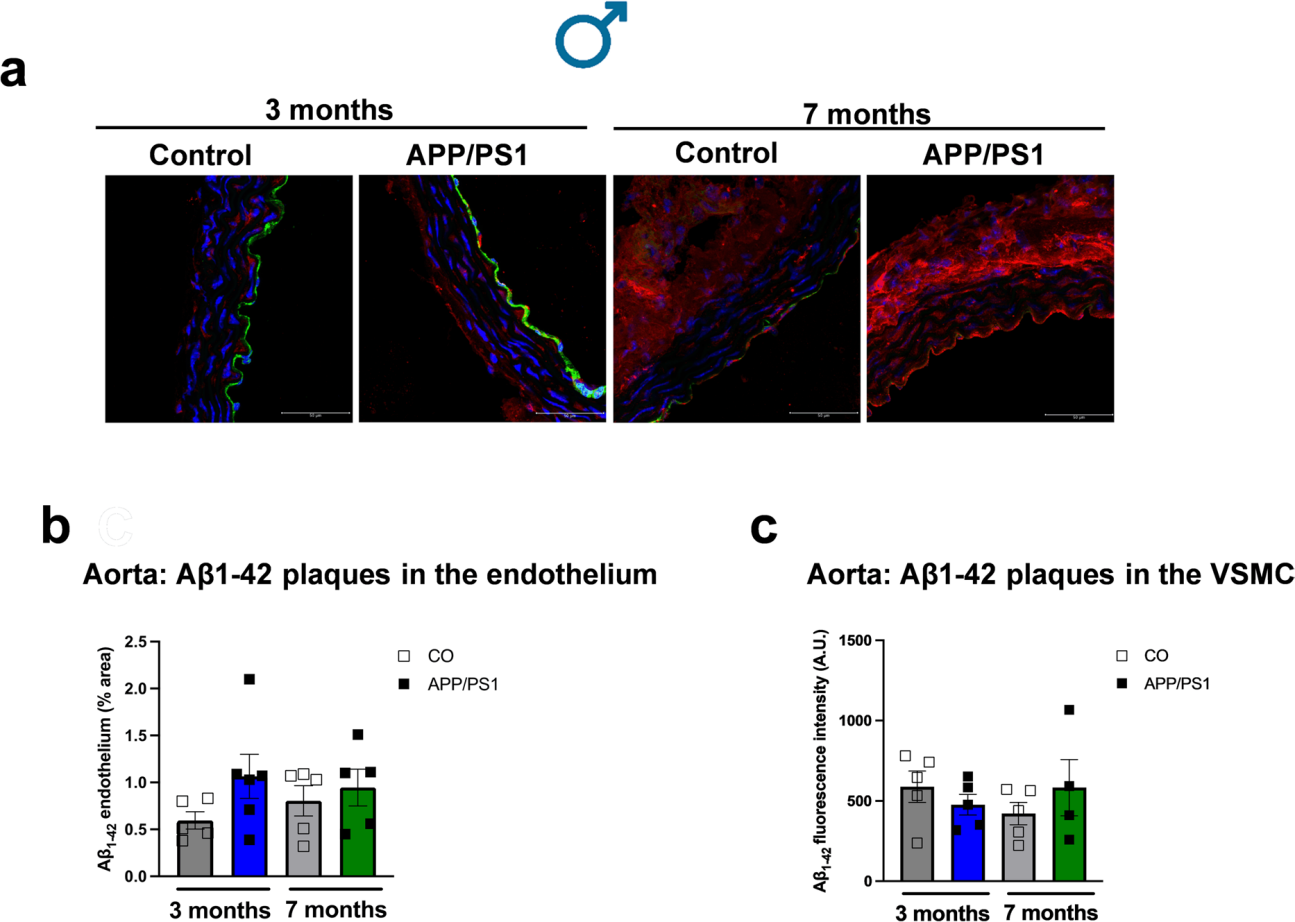
Aorta from male APP/PS1 mice did not have increased Aβ_1-42_ plaque deposition. Representative images of mice aorta showing Aβ_1-42_ plaque burden. Merged images show DAPI (blue; nuclei), Aβ_1-42_ (red), and CD31 (green) in the aortas from male APP/PS1 and C57BL/6 J mice prior to (3 months old) and during the onset of amyloid pathology (7 months old) (**a**). Bar represents 50 µm (63 × objective, 184.52 µm.^2^ area). Negative controls were aortas with secondary antibody (Alexa Fluor 647 or Alexa Fluor 488) and DAPI. Graphs show the Aβ_1-42_ quantification (% area) in the endothelium (**b**) and the vascular smooth muscle cells (VSMC, **c**). The VSMC were quantified through 10 regions of interest (70 × 72) and presented as Aβ_1-42_ fluorescence intensity (A.U.). Abbreviations: APP/PS1, Alzheimer’s disease; CO, control. Data are presented as mean ± S.E.M. Statistics: two-way ANOVA with Tukey post hoc analysis (*p* > 0.05) (male 3 month CO *n* = 5, APP/PS1 *n* = 6; male 7 month CO *n* = 5, APP/PS1 *n* = 6)

**Fig. 10:**
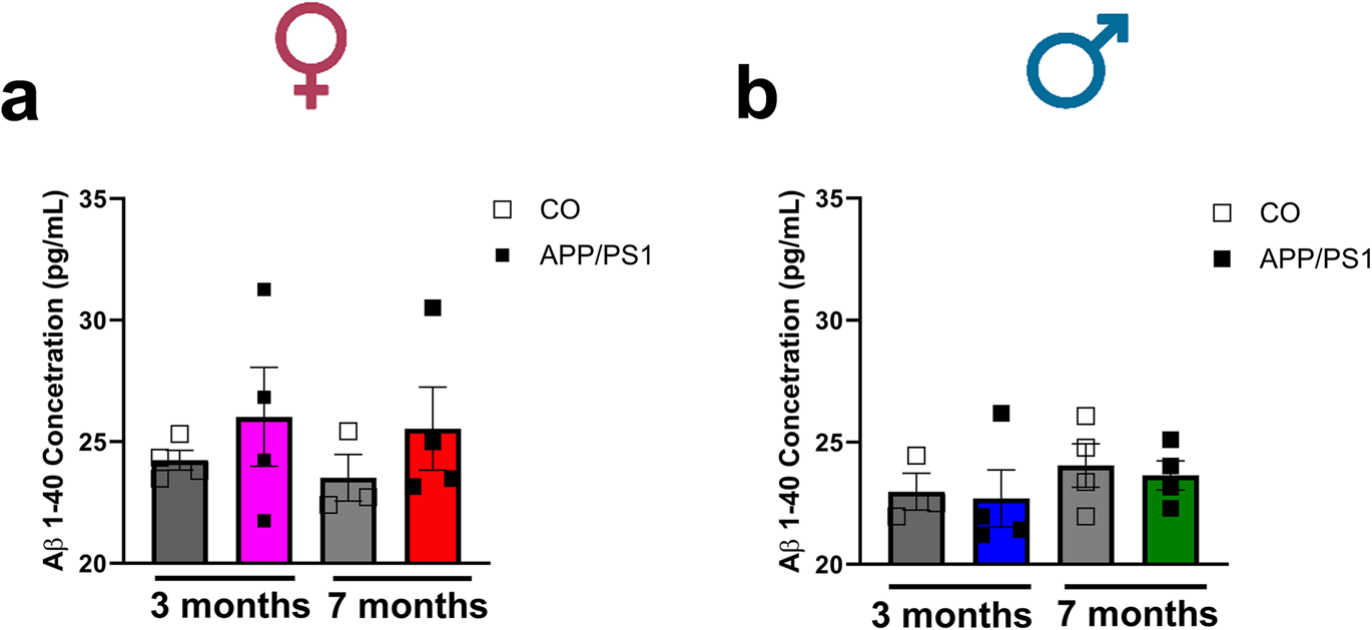
No differences were observed in the circulating Aβ_1-40_ levels in female and male APP/PS1 mice. Plasma from female (female 3 month CO *n* = 4, APP/PS1 *n* = 4; female 7 months CO *n* = 3, APP/PS1 *n* = 4) (**a**) and male (male 3 month CO *n* = 3, APP/PS1 *n* = 4; male 7 month CO *n* = 4, APP/PS1 *n* = 4) (**b**) APP/PS1 or C57BL/6 J mice prior to (3 months old) or during the onset of amyloid pathology (7 months old). Abbreviations: APP/PS1, Alzheimer’s disease; CO, control. Concentration of Aβ_1-40_ is presented as pg/mL. Data are presented as mean ± S.E.M. Statistics: two-way ANOVA with Tukey post hoc analysis (*p* > 0.05)

We performed PWV measurements on the aortic arch (Fig. 7a) to evaluate vascular stiffness prior to and during the onset of amyloid pathology. The PWV was increased in the 7-month-old female APP/PS1 group compared to the 3-month-old female APP/PS1 mice (Fig. 7b). This suggests that during the onset of amyloid pathology, females are more susceptible to develop aortic stiffness than males. It seems that the progression of the vascular dysfunction is marked with the aging of the female APP/PS1 mice as they exhibited an increased aortic stiffness compared to the female APP/PS1 mice prior to the onset of amyloid pathology. Furthermore, the 7-month-old female APP/PS1 mice had increased cardiac output and stroke volume (Fig. 7c and d) compared to the 3-month-old APP/PS1 group. On the other hand, no differences were observed in the PWV of the male control or APP/PS1 mice (Fig. 7f), or in the cardiac output or stroke volume of the male mice independent of age (Fig. 7g and h). Interestingly, we observed that during the development of amyloid pathology, only 7-month-old male APP/PS1 mice showed lower SBP values compared to the controls (Fig. 7i), but no differences were observed in the 7-month-old female APP/PS1 mice (Fig. 7e). No differences in SBP were observed in male or female mice prior to the onset of amyloid pathology (Fig. 7e and i). In an effort to understand whether Aβ deposition also occurs in the peripheral vasculature, we measured the Aβ_1-42_ plaque burden in the aorta. We observed that although Aβ_1-42_ plaques were detected in the endothelium and vascular smooth muscle layer of the aorta, they were not significantly increased in female (Fig. 8a–c) or male (Fig. 9a–c) control and APP/PS1 mice. Interestingly, the female APP/PS1 mice at 7 months of age had a reduced expression of BACE 1 (Online Resource 4b), while the 3-month-old male APP/PS1 mice had increased expression of presenilin 1 (PSEN-1) which forms the active core of the γ-secretase enzyme (Online Resource 4e). However, there were no differences observed in the levels of plasma Aβ_1-40_ regardless of sex (Fig. 10a and b).

Moreover, no changes were observed in the aorta morphometric parameters including wall thickness, wall-to-lumen ratio, and CSA values regardless of sex (Online Resource 5 and 6), as well as in the other cardiac function parameters in all the groups (Online Resource Table 1).

### Increased body weight in the APP/PS1 mice during the onset of amyloid pathology

The 7-month-old female APP/PS1 (Online Resource 7a) and the 7-month-old male APP/PS1 mice (Online Resource 7b) had an exacerbated increase in body weight when compared to controls. However, no significant differences were observed in the levels of estradiol in the 3- and 7-month-old female mice (Online Resource 7c).

## Discussion

In our present study, we sought to elucidate the contributions of the cardiovascular system to the development of AD. Although AD has long been considered to be a brain-specific neurodegenerative disease, there is increasing evidence of a direct link between AD and CVDs [2]. Our data show that prior to the onset of amyloid pathology, both male and female mice present with endothelial dysfunction, and as the disease progresses, both male and female APP/PS1 mice show resistance artery hypercontractility. This vascular dysfunction suggests the loss of a particular vascular protective factor irrespective of sex.

In the course of our study, we observed a higher mortality of the female APP/PS1 mice compared to the controls, a fact that was not present in the male APP/PS1 mice. In fact, the earliest reported death of the female APP/PS1 mice was at 9 weeks, less than 3 months of age. Our findings are in line with a previous study by Rimante and co-workers [19] reporting that APP/PS1 mice (3–4.5 months old) have increased mortality due to unprovoked seizures. Similarly, using the same mouse model, Xiong et al. observed that 35% of the female APP/PS1 mice died before the age of 6 months. The transgenes APPswe and PSEN1dE9 lead to high levels of Aβ_1-42_ that aggregate and form oligomers from an early developmental stage of the mice, triggering strong neuroinflammatory responses resulting in severe central nervous system dysfunction and premature death [20]. Although we were not able to determine the actual cause of death in our female mice, the occurrence of seizures cannot be excluded. Since we observed high levels of Aβ_1-42_ deposition in the hippocampus that could be related to neuroinflammatory signaling leading to neuronal dysfunction, it may represent a phenomenon to be investigated in future studies.

Alzheimer’s disease is characterized by difficulties with memory, language, and behavioral impairment leading to loss of cortical function and ultimately death [21]. APP/PS1 transgenic mice develop beta-amyloid deposits in the brain by 6–7 months of age [10]. These mice display Aβ deposits in two regions that play a distinctive role in recognition memory, i.e., the hippocampus and the cortex (perirhinal and frontal cortex). The hippocampus is involved in object-place memory; the cortex has the perirhinal cortex involved in novelty detection, and the frontal cortex contributes to the memory of the temporal sequence of events [22]. We therefore examined NOR memory in our mice prior to and during the onset of amyloid pathology in order to assess cognitive ability. Our data showed that APP/PS1 and control mice at 3 months of age spent similar times exploring the novel object over the familiar one, and this was observed in both male and female mice. Their ability to detect a novel stimulus would suggest that functions supported by the perirhinal cortex are not compromised, and they have an intact spatial and recognition memory prior to the onset of amyloid pathology. However, it is interesting that the male APP/PS1 mice at the age of 7 months were more susceptible to cognitive decline, but the female APP/PS1 mice at this age did not show a similar effect. A study by Van Dam and colleagues [23] used a different transgenic mouse model (APP23) at 6–8 weeks, 3 months, and 6 months of age. They observed plaque formation in the hippocampus of male mice at 6 months of age. Despite this, the mice had increased plaque-associated Aβ_1-42_ peptides and impaired spatial memory prior to the plaque formation. This finding demonstrates that neuronal and/or behavioral alterations are related to the presence of toxic soluble Aβ and the subtle changes they impart as they aggregate into oligomers, more than they are related to plaque formation [23, 24]. Similar to our findings, Bonardi et al. [25] studied this strain of female mice and observed that even at 6 months of age when they are expected to have an increased plaque burden, they had an intact object recognition memory [25]. They proposed that the lack of memory impairment could be due to the simplicity of the tasks or the short intertrial interval (ITI) [25]. There is also evidence that although the hippocampus is important in object recognition, the integrity of the entorhinal and perirhinal cortices (temporal lobe) plays a central role in this test. The Aβ load is not the only cause of cognitive impairment, but also their location in the brain and the response in those areas [26].

On the other hand, although the 7 months old female APP/PS1 mice in our study did not exhibit significant cognitive decline, they had a significant increase in the hippocampus Aβ_1-42_ plaque burden. Interestingly, the plaques were specifically colocalized with CD31 which is a marker of endothelial cells, indicating that the plaques were deposited on the intima layer of the blood vessels, affecting the endothelial cells. Although there is a likelihood of the development of cerebral amyloid angiopathy, it has been shown to be propagated mostly by the accumulation of Aβ_1-40_ rather than Aβ_1-42_ in the vascular, perivascular, and extracellular matrix of blood vessels [27]. Our study, however, shows that the Aβ_1-42_ plaques which are known to accumulate in the brain parenchyma in AD can also be deposited in the blood vessels in a sex-dependent manner, with the female APP/PS1 mice at the age of 7 months showing higher deposition than the male APP/PS1 mice. Indeed, AD has been shown to be more prevalent in women than men with increasing age [1]. Female APP/PS1 mice have been shown to have elevated Aβ_1-42_ peptides, with the highest amounts being detected in the cortices and the hippocampus [20, 28]. Furthermore, there is a significant role played by sex differences in the initiation, progression, and clinical manifestation of AD [29]. Estrogen is regulated by the nuclear receptors, estrogen receptor alpha (ERα) and estrogen receptor beta (ERβ) [30], which are broadly distributed in the central nervous system (CNS) [31]. We did not observe any differences in the serum estradiol levels between female APP/PS1 and control mice. However, we did not evaluate the hippocampus estrogen levels and estrogen receptor expression, which could be associated with the sex differences observed in the present study.

Different studies have shown a link between vascular diseases and AD whereby high concentrations of Aβ peptides are toxic to the brain and peripheral endothelial cells and cause cellular damage, enhanced vasoconstriction and impair endothelium-dependent relaxation [2, 9]. Peripheral microvessels are important to evaluate since any changes in vascular function during homeostasis, such as increase or decrease in vascular contraction and/or increase or decrease in vasodilation, may lead to chronic and irreversible damage in the cerebral autoregulation resulting in accelerated progression of AD. Our data showed an interesting sex-independent response whereby the male and female APP/PS1 mice had a marked endothelial dysfunction prior to the onset of amyloid pathology. This result is in line with a study by Iadecola and colleagues [32] who demonstrated that APP overexpression in vivo leads to a profound and selective alteration in endothelial vascular regulation. The authors showed that the altered regulation was mediated by the in vivo production of reactive oxygen species (ROS), and similar effects were shown in other in vitro studies with short-term incubations using synthetic Aβ peptides on isolated rat aorta [9, 33]. In addition, in vitro studies using isolated basilar arteries with short-term [34] or long-term [35] synthetic Aβ peptides treatment reported that the endothelial dysfunction was associated with enhanced endothelial nitric oxide synthase (eNOS) phosphorylation in the inhibitory residues threonine 495 and serine 116 [36, 37], and reduced ACh-induced phosphorylation on serine 1177. They also showed that these effects were due to the altered function of the endothelial cells rather than damage and/or apoptosis of the endothelial layer [35]. Our study expands the existing data as we used isolated peripheral mesenteric resistance arteries (MRA) from a mouse model of AD that co-express the mutated Swedish APP gene and the exon-9 deleted variant of the PS1 gene thus increasing the amyloid peptide burden. Surprisingly, during the onset of amyloid pathology, the male and female mice showed a restoration of the endothelium-dependent relaxation. Here, we were not able to elucidate which factor is related to the endothelial function “improvement” or compensation in the APP/PS1 mice at 7 months of age. Whether it is related to an adaptation of the microvascular environment to the disease condition, a “functional catch-up mechanism,” or is due to a specific activation of intracellular signaling, such as vasodilator inflammatory mediators such as hydrogen peroxide [38], is still to be determined.

Evidence shows that Aβ peptides enhance the vasoconstriction of rat aortas to PE or endothelin-1 [9, 33, 39]. In our study, female APP/PS1 mice at 3 months of age had a decreased receptor-independent contraction to KCl prior to the onset of amyloid pathology. However, the arteries from the male and female mice showed enhanced hypercontractility to both agonist-dependent (e.g., PE and U46619) and independent (KCl) contractile factors during the onset of amyloid pathology. Similar results of enhanced U46619 contractile effect were observed in cerebral microvessels of mice overexpressing the amyloid precursor protein [32], suggesting an inflammatory activation induced by this agent. It is important to highlight that both agonists PE and U46619, through different receptor-mediated responses, and also the receptor-independent contraction mediated by high KCl levels, were able to promote vascular hypercontractility during the onset of amyloid pathology, suggesting that it can be a general vascular effect triggered by the AD development and not an exclusive G-protein coupled receptor (GPCR) response. In addition, there were no differences in the collagen or elastin of the mesenteric resistance arteries of these mice regardless of age and sex. This suggests that the hypocontractility (low contraction) observed in the arteries from the young animals may be due to changes in the vascular smooth muscle cells (VSMC) [e.g., calcium, hypotrophic remodeling (loss of muscle) phenotype, or other mechanisms]. Whether this increased peripheral vascular response is also related to enhanced inflammation due to peripheral Aβ deposition in MRA or by a central mechanism that leads to a systemic inflammation needs further investigation.

Vascular remodeling with arterial stiffness is a hallmark in CVD, including hypertension, and evidence associates hypertension with rapid cognitive decline and neuroanatomical changes in AD [2, 40]. Our data revealed increased PWV as well as cardiac output and stroke volume in the female APP/PS1 mice at 7 months of age compared to those at 3 months of age, with no changes in the PWV of the male mice. This suggests that during the onset of amyloid pathology, females were more susceptible to develop aortic stiffness than males. In addition, the vascular dysfunction is marked with the aging of the female APP/PS1 mice as they showed an increased aortic stiffness at the age of 7 months compared to 3 months. This is in line with a study which showed that arterial stiffness increases with age and is an independent predictor of progressive Aβ deposition, and can be an eventual factor linking hypertension and cognitive impairment [41]. The male APP/PS1 mice seem to have a slower progression of the arterial stiffness as they did not show any differences based on their age or the presence of the disease.

While hypertension has been linked with increased AD risk, there is substantial evidence showing that low blood pressure late in life is also related to AD and cognitive impairment [42]. Our data illustrate this link and are further strengthened by the fact that the male APP/PS1 mice at 7 months of age not only had lower SBP values, but also poor recognition of the novel object indicating that they were experiencing cognitive decline. Interestingly, no SBP differences were observed in the female APP/PS1 mice prior to or during the onset of amyloid pathology. However, species-level differences as well as genetic and epigenetic modulation of the cardiovascular system in human AD may differ from this particular EOAD mouse strain.

Finally, although Aβ deposition has been shown to occur both in the brain and in peripheral tissues [27, 43], our study was not able to show increased deposition of Aβ_1-42_ in the aortas mostly because this isoform of Aβ is more dominant in the brain. Furthermore, the plasma levels of Aβ_1-40_ which is the dominant isoform in the periphery were not significantly changed. Some of the proposed mechanisms for this include Aβ peptides having a short half-life after secretion into plasma as well as binding to Aβ peptide-binding proteins like albumin and lipoproteins which inhibit its polymerization [27].

Our study was conducted in 7-month-old APP/PS1 mice which is the age described to be the lower limit of AD features including Aβ deposition in this mouse strain of EOAD [10, 11]. This could represent a limitation of our study, since at an older age (i.e., > 1 year), they could present a more severe characteristic of cognitive decline [20], and this could be associated with more pronounced vascular remodeling in conductance arteries. Of particular importance is highlighting that these transgenic mice models only simulate certain aspects of Alzheimer’s disease, and the phenotypes exhibited by these mice might not fully represent the broader range of clinical conditions observed in humans with Alzheimer’s disease. At this moment, our study was not able to identify which factor(s) could be responsible for the sex-specific influences of the vascular pathology to AD progression. Whether any hormonal levels could impact the development of the AD and affect the cardiovascular system in this mouse strain remains unclear and should be the focus of future studies.

In the present study, we did not investigate the cause of body weight fluctuations associated with AD as this was outside the scope of our study. However, it is important to emphasize that obesity has been associated with an increased risk of dementia with several studies reporting a reverse causality, with weight loss preceding the onset of dementia [44, 45]. For instance, it was recently observed that while patterns of decline in body mass index (BMI) were associated with dementia, a subgroup with a pattern of initial increasing BMI followed by declining BMI, both occurring within mid-life, appeared to be central to declining BMI–dementia association [45, 46]. This highlights that further validations are needed to provide any association or cause-effect mechanism of this phenomenon.

Overall, our data show that male and female APP/PS1 mice present with vascular dysfunction that occurs prior to the onset of amyloid pathology. In addition, there were significant sex differences in the blood pressure levels, arterial stiffness, and the Aβ_1-42_ plaque deposition and colocalization with endothelial cells in the hippocampus during the onset of amyloid pathology. Moreover, our study evidenced that the males have found a way to compensate and protect against overall increases in mortality, and the females are less likely to be diagnosed because they do not present with overt cognitive dysfunction. Although early diagnosis of AD symptoms in the clinical setting poses a challenge due to the invasive nature of the procedure, our study results show that both male and female mice present with endothelial dysfunction prior to the amyloid pathology. Based on this and other factors, perhaps including a screening protocol for endothelial function in peripheral arteries (e.g., brachial artery flow-mediated dilation) as a preventative strategy for patients who have a history of familial AD or are in the early stages of diagnosis could be a good clinical strategy in the future. Overall, our study plays a critical role in highlighting the link between these two highly prevalent pathologies, AD and CVD, by demonstrating that peripheral vascular abnormalities may be an early marker and potential mediator of EOAD. However, exacerbated aortic stiffness and pressure pulsatility during the onset of amyloid pathology may be associated with a greater burden of Aβ formation in endothelial cells in the hippocampus from female but not male APP/PS1 mice.

## Supplementary Materials

Below is the link to the electronic supplementary material.

ESM 1 (PNG 253 kb)

High resolution image (TIF 1618 kb)

ESM 2 (PNG 622 kb)

High resolution image (TIF 4113 kb)

ESM 3 (PNG 662 kb)

High resolution image (TIF 6.03 mb)

ESM 4 (PNG 2176 kb)

High resolution image (TIF 12520 kb)

ESM 5 (PNG 2262 kb)

High resolution image (TIF 9582 kb)

ESM 6 (PNG 129 kb)

High resolution image (TIF 1425 kb)

ESM 7 (DOCX 27 kb)

## Data Availability

Further information and requests for resources and reagents should be directed to and will be fulfilled by the corresponding author, Camilla F. Wenceslau, PhD (Camilla.Wenceslau@uscmed.sc.edu).

## Abbreviations

*APP/PS1*: Alzheimer’s disease
*PWV*: Pulse wave velocity
*Aβ*: Amyloid beta peptide. Graphical abstract was created with BioRender.com
