# Supplementary figures and images for: Vascular dysfunction occurs prior to the onset of amyloid pathology and Aβ plaque deposits colocalize with endothelial cells in the hippocampus of female APPswe/PSEN1dE9 mice
Source: GeroScience. 2024 Jun 11;46(6):5517–36. doi: 10.1007/s11357-024-01213-0 (PMC11493946; doi:10.1007/s11357-024-01213-0)

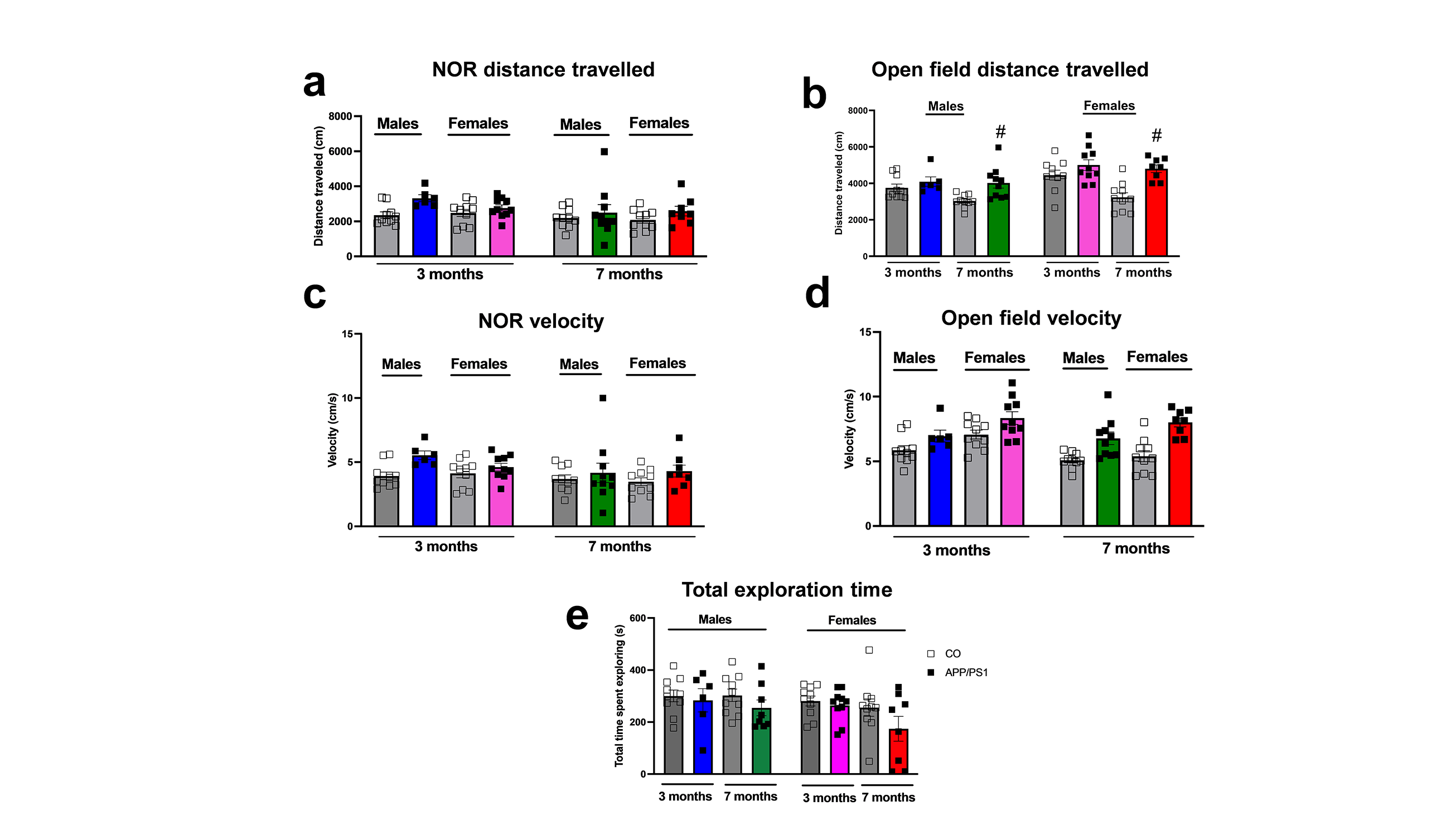

Supplement: Supplementary file 1 — (PNG 253 kb) [file 11357_2024_1213_Fig11_ESM.png]

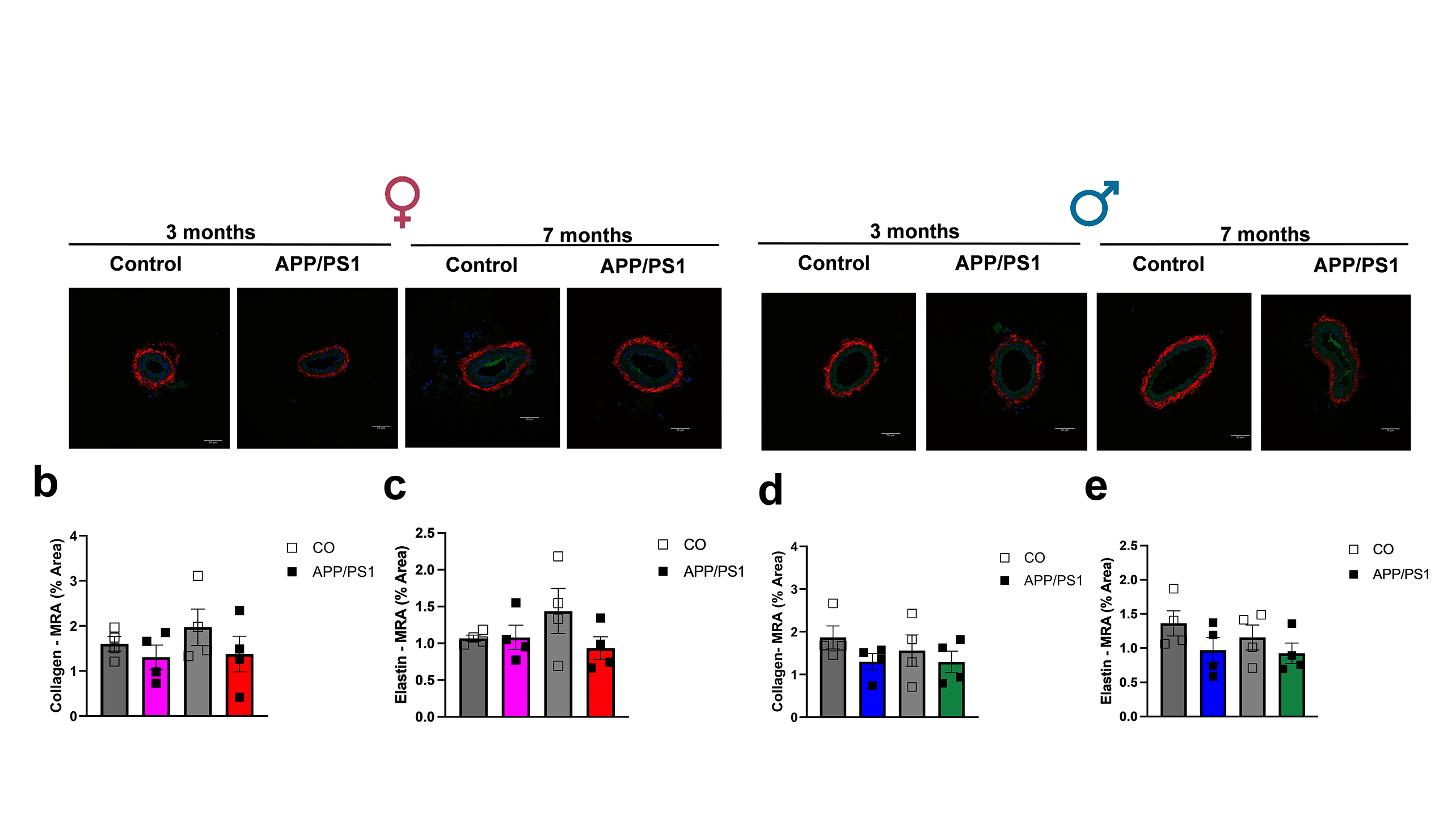

Supplement: Supplementary file 3 — (PNG 622 kb) [file 11357_2024_1213_Fig12_ESM.png]

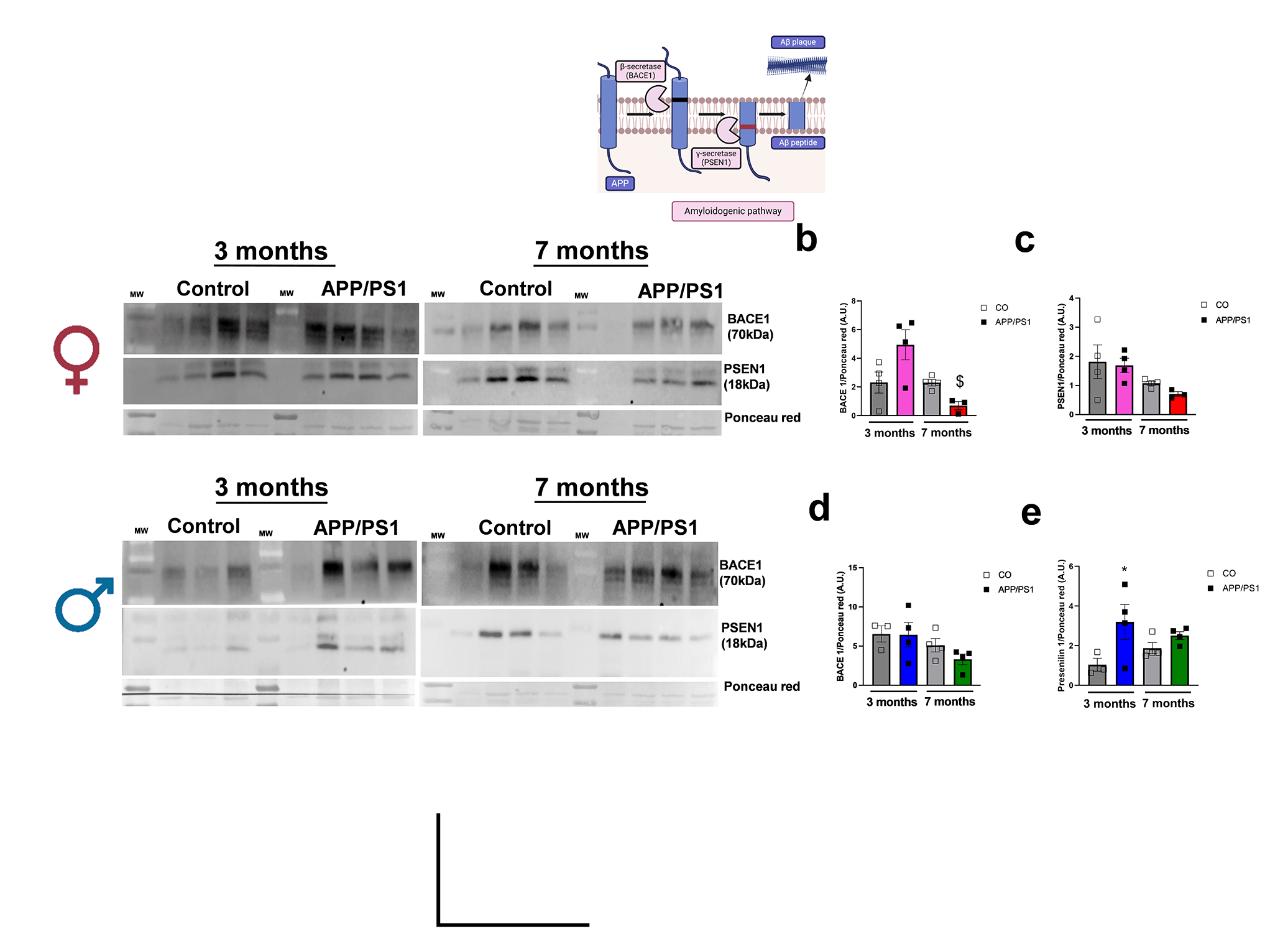

Supplement: Supplementary file 5 — (PNG 662 kb) [file 11357_2024_1213_Fig13_ESM.png]

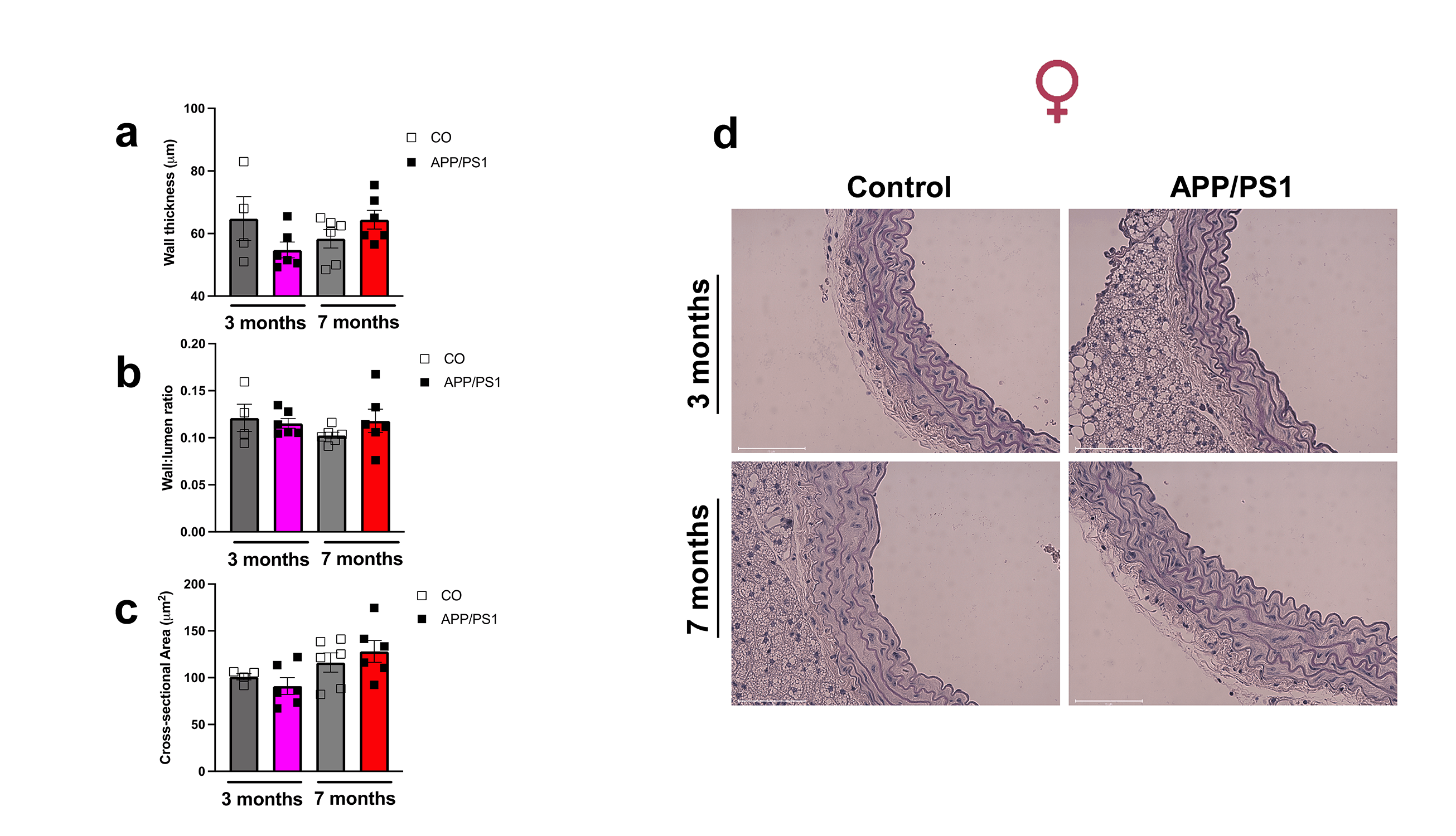

Supplement: Supplementary file 7 — (PNG 2176 kb) [file 11357_2024_1213_Fig14_ESM.png]

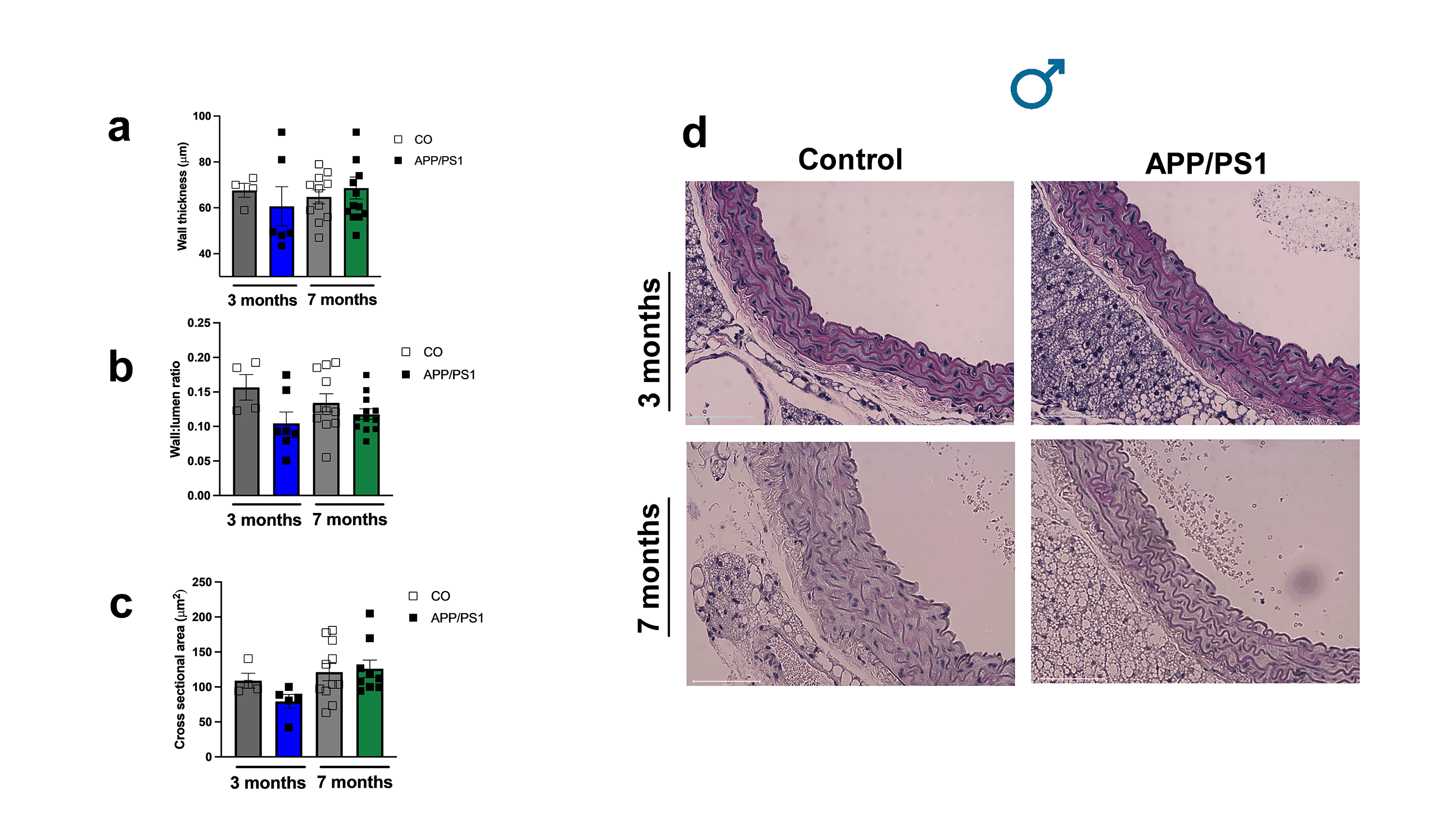

Supplement: Supplementary file 9 — (PNG 2262 kb) [file 11357_2024_1213_Fig15_ESM.png]

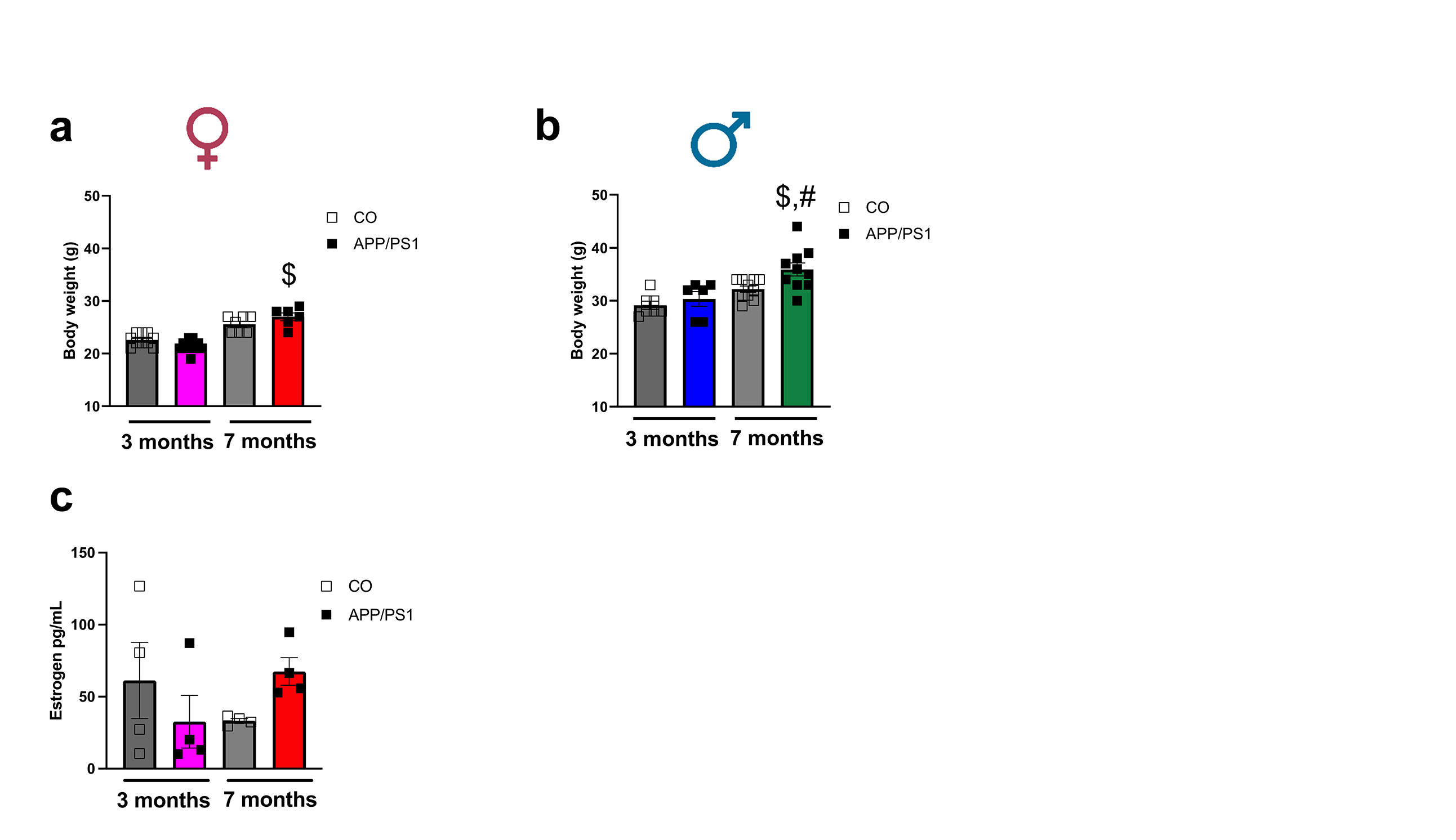

Supplement: Supplementary file 11 — (PNG 129 kb) [file 11357_2024_1213_Fig16_ESM.png]
