# Supplementary material for: Vascular dysfunction occurs prior to the onset of amyloid pathology and Aβ plaque deposits colocalize with endothelial cells in the hippocampus of female APPswe/PSEN1dE9 mice
Source: GeroScience. 2024 Jun 11;46(6):5517–36. doi: 10.1007/s11357-024-01213-0 (PMC11493946; doi:10.1007/s11357-024-01213-0)
Supplement: Supplementary file 13 — (DOCX 27 kb) [file 11357_2024_1213_MOESM7_ESM.docx]

| **Parameters (units)** | **CO - 3 months** | **APP/PS1 – 3 months** | **CO- 7 months** | **APP/PS1- 7 months** | **CO- 3 months** | **APP/PS1- 3 months** | **CO- 7 months** | **APP/PS1- 7 months** |
| --- | --- | --- | --- | --- | --- | --- | --- | --- |
| **Heart rate (bpm)** | 403.27 ± 24.85, n=6 | 435.61 ± 8.90, n=6 | 469.37 ± 9.78, n=9 | 464.18 ± 25.22, n=9 | 466.06 ± 5.61, n=10 | 455.25 ± 14.97, n=10 | 452.34 ± 18.79, n=6 | 510.84 ± 65.00, n=7 |
| **Ejection fraction (%)** | 69.53 ± 4.58, n=6 | 69.20 ± 3.86, n=6 | 59.00 ± 4.88, n=9 | 67.20 ± 2.67, n=9 | 71.30 ± 33.06, n=10 | 67.12 ±3.22, n=10 | 71.75 ± 2.26, n=6 | 66.74 ± 4.66, n=7 |
| **Fractional shortening (%)** | 39.01 ± 3.72, n=6 | 38.75 ± 3.23, n=6 | 31.72 ± 3.50, n=9 | 36.84 ± 2.02, n=9 | 40.34 ± 2.57, n=10 | 36.68 ± 2.34, n=10 | 40 ± 1.96, n=6 | 36.88 ± 3.27, n=7 |
| **LV mass (mg)** | 97.99 ± 14.73, n=6 | 125.45 ± 11.92, n=6 | 131.67 ± 10.89, n=9 | 137.54 ± 6.07, n=9 | 70.10 ± 5.39, n=10 | 82.58 ± 11.63, n=10 | 99.42 ± 11.23, n=6 | 98.09 ± 10.40, n=7 |
| **LVAW systole (mm)** | 1.26 ± 0.08, n=6 | 1.35 ± 0.02, n=6 | 1.17 ± 0.07, n=9 | 1.34 ± 0.08, n=9 | 1.09 ± 0.06, n=10 | 1.09 ± 0.04, n=10 | 1.15 ± 0.18, n=6 | 1.13 ± 0.12, n=7 |
| **LVAW diastole (mm)** | 0.84 ± 0.07, n=6 | 0.86 ± 0.03, n=6 | 0.83 ± 0.03, n=9 | 0.93 ± 0.07, n=9 | 0.64 ± 0.05, n=10 | 0.72 ± 0.04, n=10 | 0.82 ± 0.14, n=6 | 0.81 ± 0.08, n=7 |
| **LVPW systole (mm)** | 1.37 ± 0.18, n=6 | 1.53 ± 0.17, n=6 | 1.36 ± 0.15, n=9 | 1.57 ± 0.14, n=9 | 1.13 ± 0.09, n=10 | 1.31 ± 0.14, n=10 | 1.46 ± 0.12, n=6 | 1.21 ± 0.13, n=7 |
| **LVPW diastole (mm)** | 0.84 ± 0.23, n=6 | 1.00 ± 0.18, n=6 | 0.99 ± 0.14, n=9 | 1.13 ± 0.18, n=9 | 0.69 ± 0.07, n=10 | 0.89 ± 0.17, n=10 | 1.04 ± 0.14, n=6 | 0.78 ± 0.14, n=7 |
| **Tibia length (cm)** | 1.77 ± 0.05, n=7 | 1.85 ± 0.06, n=6 | 1.91 ± 0.03, n=10 | 1.87 ± 0.02, n=10 | 1.75 ±0.03, n=10 | 1.72 ± 0.04, n=10 | 1.89 ± 0.03, n=7 | 1.92 ± 0.05, n=6^$^ |
| **Uterus weights** |  |  |  |  | 0.08 ± 0.01, n=6 | 0.09 ± 0.02, n=9 | 0.10 ± 0.02, n=7 | 0.09 ± 0.01, n=6 |

**Online Resource Table 1**. No differences observed in the cardiac function of male and female APP/PS1 mice.


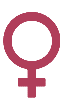


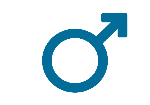


^$^ Different from 3 months old female APP/PS1 (Two-way ANOVA; Tukey post-hoc; p<0.05).
